# Direct assessment of confinement effect in zeolite-encapsulated subnanometric metal species

**DOI:** 10.1038/s41467-022-28356-y

**Published:** 2022-02-10

**Authors:** Lichen Liu, Miguel Lopez-Haro, Jose Antonio Perez-Omil, Mercedes Boronat, Jose J. Calvino, Avelino Corma

**Affiliations:** 1 Instituto de Tecnología Química, Universitat Politècnica de València-Consejo Superior de Investigaciones Científicas, Av. de los Naranjos s/n, Valencia, 46022 Spain; 2 Department of Chemistry, Tsinghua University, Beijing, 100084 China; 3 Departamento de Ciencia de los Materiales e Ingeniería Metalúrgica y Química Inorgánica, Facultad de Ciencias, Universidad de Cádiz, Cádiz, Spain

**Keywords:** Heterogeneous catalysis, Porous materials

## Abstract

Subnanometric metal species confined inside the microporous channels/cavities of zeolites have been demonstrated as stable and efficient catalysts. The confinement interaction between the metal species and zeolite framework has been proposed to play the key role for stabilization, though the confinement interaction is elusive to be identified and measured. By combining theoretical calculations, imaging simulation and experimental measurements based on the scanning transmission electron microscopy-integrated differential phase contrast imaging technique, we have studied the location and coordination environment of isolated iridium atoms and clusters confined in zeolite. The image analysis results indicate that the local strain is intimately related to the strength of metal-zeolite interaction and a good correlation is found between the zeolite deformation energy, the charge state of the iridium species and the local absolute strain. The direct observation of confinement with subnanometric metal species encapsulated in zeolites provides insights to understand their structural features and catalytic consequences.

## Abstract (web summary)

Zeolite-encapsulated metal nanoparticles have important catalytic properties, but their effect on the zeolite local structure has been difficult to characterize. Here the authors, using DFT calculations and scanning transmission electron microscopy, characterize the local strain due to confinement effects in metal-zeolite catalysts.

## Introduction

Confinement effects have been widely recognized in catalysis, which are related to the restricted geometric or electronic structure of the active site and/or substrate molecule during the catalytic cycles^1^. In the field of heterogeneous catalysis based on solid materials, confinement effects are usually associated with nanoparticles located in a small space or interfacial structure, leading to restricted mobility and structural flexibility^2^. Under some circumstance, it is possible to generate or stabilize unfeasible active sites and/or transition states by locating them in a constrained environment. Consequently, unique reactivity and selectivity can be achieved, as widely demonstrated with zeolite-based catalysts^3–5^ and metal catalysts immobilized in microporous and mesoporous materials^6–8^.

One typical exhibition of confinement effect is the greatly enhanced stability of encapsulated metal species inside porous materials, such as zeolites^9–12^. For instance, small metal nanoparticles or subnanometric metal clusters can resist against sintering under high-temperature conditions when they are encapsulated in the cavities or channels of the zeolite frameworks^13^. The mobility of the tiny metal particles will be greatly restricted by the rigid zeolite frameworks, resulting in high thermal stability against sintering, even in reductive atmosphere^14,15^. From a structural point of view, the constrain imposed on the encapsulated metal particle by the zeolite framework may also cause local structural changes in the zeolite framework due to the counterforce, if the metal-zeolite interaction is strong enough. However, this potential influence has not been observed because the structural changes of the zeolite framework will be too subtle to be captured by averaging techniques such as X-ray diffraction, solid-state NMR or IR spectroscopy due to the low metal loadings (≤2 wt%) in commonly used metal-zeolite catalysts.

Lattice strain, considered as atomic-level crystalline distortion with respect to the perfect crystalline structure, is widely present in various nanomaterials, such as metallic nanoparticles, oxides, and carbon nanotubes^16–18^. The induction of lattice strain can be caused by the imperfect local structures, such as the defective sites in the crystallite or atomic displacement at surface region. Lattice strain can also be caused by the interfacial interaction at the boundary of two different materials, as observed with supported metal catalysts and hybrid nanomaterials^19,20^. In conventional supported metal catalyst, the sites with higher lattice strain are proposed as more reactive sites to interact with substrate molecules^21,22^. In this context, considering a more intimate contact between the encapsulated metal species and the zeolite framework, lattice strain may also be present in metal-zeolite materials, though it has not been directly measured yet, though metal-zeolite materials have been intensively studied by various techniques^23,24^.

In this work, we have studied the interaction between encapsulated metal species and zeolite framework by DFT calculations and then carried out the direct measurement of local strain in zeolite structure by the scanning transmission electron microscopy-integrated differential phase contrast (STEM-iDPC) imaging technique^25^, which indicates a direct observation of confinement effect in metal-zeolite catalysts.

## Results

### Structures of the confined metal species

To understand the structural features of metal species encapsulated in the microporous environment of zeolite structure and to investigate the potential structural changes caused by the metal-zeolite interaction, we have carried out theoretical studies by DFT-D calculations with Ir species encapsulated in pure-silica MWW zeolite, which comprises bi-dimensional sinusoidal 10-membered ring (10MR) channels and 12-membered ring (12MR) supercages interconnected by 10MR windows, as the model material (see Supplementary Fig. S1 for more descriptions)^26^.

Initially, Ir single atoms (Ir_1_) were placed at different positions within the MWW framework to figure out their preferential position in the zeolite structure (see models in Supplementary Fig. 2). Analysis of the optimized geometries shown in Supplementary Fig. 3 and of the energies and charges given in Table 1 indicates the existence of two ways of interaction of neutral Ir_1_ atoms with neutral silica frameworks. On one hand, a Si–O bond of the small 5MR or 6MR rings is broken to form of a new Ir–Si bond with an optimized bond length of 2.23–2.24 Å and two new O–Ir bonds that greatly stabilize the Ir atom (Ir_1_–A to Ir_1_–D configurations), whose calculated encapsulation energies are around −200 kJ/mol (see Table 1)^27^.The formation of the Ir–Si bond results in a net charge transfer to the Ir_1_ atom, that becomes negatively charged by −0.5 electrons, and decreases the number of unpaired electrons from three in vacuum to only one (see Table 1). On the other hand, the Ir_1_ atom can be stabilized in the 6MR of the double 6-member ring (D6R) unit as in structure Ir_1_–E, or in the larger 10MR rings (Ir_1_–F and Ir_1_–G). In these systems, the Ir_1_ atom is stabilized only through weaker Ir–O interactions with the framework oxygen atoms, which explains their high relative instability and lower encapsulation energy values (see Table 1). The charge transfer to Ir_1_ is negligible, and the atom maintains three unpaired electrons as in vacuum.

**Table 1:** DFT calculated relative (E_rel_), encapsulation (E_enc_) and deformation (E_def_) energies (in kJ/mol), net atomic charges on Ir_n_ atoms and clusters (qIr_n_ in e) and number of unpaired electorns (M) for the twenty-four Ir@MWW structures investigated in this work.

Site	Location	E_rel_ (kJ/mol)	E_enc_ (kJ/mol)	E_def_ (kJ/mol)	qIr_n_ (e)	M
Ir_1_–A	5MR in channel	0	−247	521	−0.526	1
Ir_1_–B	5MR in cage	32	−215	513	−0.528	1
Ir_1_–C	5MR in window	67	−180	529	−0.490	1
Ir_1_–D	6MR in cage	36	−211	525	−0.521	1
Ir_1_–E	D6R	98	−149	59	0.016	1
Ir_1_–F	10MR window	162	−84	1	0.041	3
Ir_1_–G	10MR channel	154	−92	5	0.042	3
Ir_4_–H	10MR channel (sq)^a^	0	−312	43	0.066	2
Ir_4_–I	5MR in channel	45	−267	557	−0.451	2
Ir_4_–J	10MR channel (rh)^b^	119	−193	64	0.083	2
Ir_4_–K	10MR window (sq)^a^	59	−253	49	0.054	2
Ir_4_–L	10MR window (sq)^a^	29	−283	160	−0.074	4
Ir_4_–M	10MR window (rh)^b^	79	−233	280	−0.232	4
Ir_4_–N	supercage (sq)^a^	51	−261	150	−0.010	2
Ir_4_–O	supercage	128	−184	208	−0.084	2
Ir_4_–P	6MR in supercage	201	−111	582	−0.529	4
Ir_4_–Q	supercage (sq)^a^	30	−282	64	0.061	2
Ir_4_–R	supercage (rh)^b^	95	−217	46	0.042	2
Ir_4_–S	D6R	99	−213	1632	−1.504	2
Ir_13_–T	supercage	194	−458	16	0.068	5
Ir_13_–U	supercage	74	−578	647	0.656	5
Ir_13_–V	supercage	0	−651	43	0.174	5
Ir_13_–W	10MR channel	200	−452	1064	−0.891	3
Ir_13_–X	10MR window	445	−206	2268	−2.174	3

^a^Square configuration.

^b^Rhombus configuration.

To quantify the structural changes within the zeolite framework due to the incorporation of Ir_1_ atoms, we have calculated the deformation energy (E_def_) as described in the computational details section. As expected, the E_def_ values associated to broken Si–O bonds in the various structures are very large (>500 kJ/mol in all cases), but they are compensated by the formation of the new Ir–Si and Ir–O bonds during the hydrothermal synthesis or post-synthesis high-temperature treatments. As a consequence, the global process is energetically very favorable. In contrast, the incorporation of an Ir atom to the larger cavities (10MR channel and window) leads to small modifications in the SiO_2_ framework, as reflected in small deformation energies below 60 kJ/mol and negligible stabilization provided by such weak interactions. Therefore, it is inferred that isolated Ir_1_ atoms will be preferentially located in the partially broken 5MR rings of the MWW framework.

**Fig. 1:**
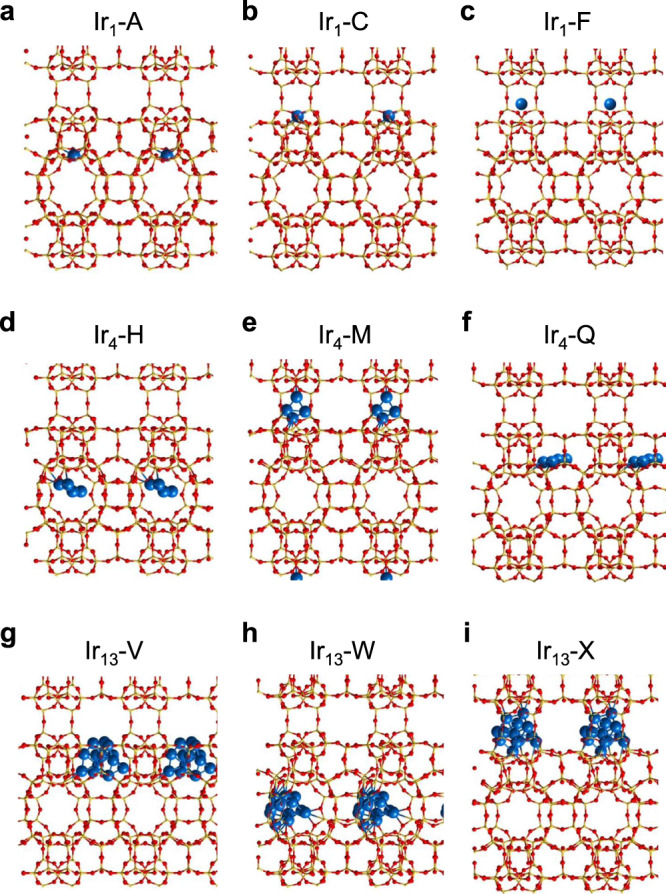
Geometries of subnanometric Ir species confined in MWW zeolite. **a** Isolated Ir atom at the 5MR of the 10MR channel, **b** isolated Ir atom at the 5 member-ring (5MR) unit in the 10 member-ring (10MR) window, **c** isolated Ir atom at the center of the 10MR window, **d** square Ir_4_ cluster at the 10MR channel, **e** Rhombus Ir_4_ cluster at the 10MR window, **f** square Ir_4_ cluster at the supercage, **g** Ir_13_ cluster at the supercage, **h** Ir_13_ cluster at the 10MR channel and **i** Ir_13_ cluster at the 10MR window. In these models, the O atoms in are presented as red spheres and Si atoms are presented as yellow spheres.

Furthermore, we have built Ir_4_ clusters to study the structural features of the confined metal clusters in MWW zeolite. According to preliminary search (see Supplementary Fig. 4 and related discussion), the Ir_4_@MWW models are established by adding three Ir atoms to the optimized structures of Ir_1_–A to Ir_1_–E. Only in two cases (structures Ir_4_–I and Ir_4_–P in Supplementary Fig. 5), one Ir atom of the Ir_4_ cluster remains inserted into the 5MR or 6MR units, while the other three Ir atoms move to form a pyramidal cluster in Ir_4_–I and a kind of linear structure in Ir_4_–P structure. Also in structure Ir_4_–S with the metal cluster inserted in the D6R unit, some Si–O bonds remain broken. In all other cases, the geometry optimization restores the Si–O bond and displaces the Ir_4_ cluster towards the closest channel or cavity. But, in contrast with what has been found for the isolated Ir_1_ atoms, the Ir_4_@MWW structures with broken Si–O bonds are not the most stable, and lay between 45 and 200 kJ/mol higher in energy than the global minimum for this system. The calculations summarized in Table 1 indicate that the most stable isomer of Ir_4_ in MWW zeolite is structure Ir_4_–H, corresponding to a square planar Ir_4_ cluster placed in the 10MR channel system and stabilized by two Ir–O bonds with the framework oxygen atoms (see Fig. 1 and Supplementary Fig. 5).

The deformation energies E_def_ calculated for the Ir_4_@MWW structures involving broken Si–O bonds in 5MR and 6MR rings are similar to those discussed for the Ir_1_@MWW cases (>500 kJ/mol), and markedly high in the case of Ir_4_–S structure (1632 kJ/mol). The destabilization effect cannot always be compensated by the formation of Ir–Si and Ir–O bonds, resulting in relatively low encapsulation energies except for Ir_4_–I structure. Large deformations of the SiO_2_ framework are also associated to the presence of Ir_4_ clusters with distorted rhombus geometry in the supercages or in the 10MR windows connecting the supercages, with E_def_ values larger than 200 kJ/mol in some cases. Square planar Ir_4_ clusters in the 10MR channels or in the supercages exhibit the most exothermic encapsulation energy values, due to the formation of two stable Ir–O bonds combined with a low distortion of the SiO_2_ framework. The charge transfer associated to these Ir–O interactions is almost negligible, and only in the few systems with broken Si–O bonds, the Ir_4_ cluster becomes negatively charged. This is reflected in a linear relationship between the charge transferred to Ir and the deformation energy E_def_ (see Supplementary Fig. 6).

The geometry optimization of a cuboctahedral Ir_13_ particle in vacuum yielded an irregular cluster that was placed in five different positions within the pure-silica MWW framework, one in the center of the supercage close to the 10MR window, two at the narrow ends of the supercage, one in the 10MR channel and another one in the 10MR windows connecting the supercages (see Supplementary Fig. 7). Due to its large size, the Ir_13_ cluster cannot be easily accommodated in the 10MR rings and some Si–O bonds are broken, resulting in deformation energies E_def_ higher than 1000 kJ/mol for structures Ir_13_–W and Ir_13_–X. In these two cases, the formation of new Ir–Si bonds facilitates the charge transfer that makes Ir_13_ clusters to become negatively charged. A large E_def_ value of 647 kJ/mol is also found for structure Ir_13_–U with the cluster located at the narrow end of the supercage, due again to the rupture of a Si–O bond of the zeolite framework, but in this case the total charge transfer takes place in the opposite sense and the Ir_13_ particle becomes positively charged. The Ir_13_ particle can also occupy positions in the wider regions of the supercage without constraining the SiO_2_ framework. This is the case of the most stable structure Ir_13_–V, with the Ir_13_ cluster somewhat displaced from the center of the supercage, with a low charge transfer and with the SiO_2_ framework hardly strained. Notice that for symmetry reasons the amount of Ir_13_ clusters in these models is clearly larger than in the real Ir@MWW samples, and therefore the deformation energies might be overestimated. These calculation results suggest a general tendency on the influence of the location of Ir species on the metal-zeolite interaction.

### Methodology for measuring the strain of metal-zeolite material

**Fig. 2:**
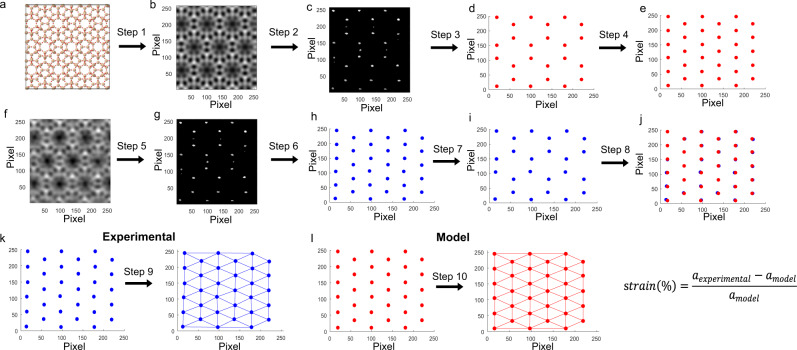
Workflow for the measurement of local strain in metal-zeolite material. **a** Model of MWW zeolite along [001] orientation. The model was scaled to the experimental image using the pixel size of the experimental image. **b** Simulated iDPC image according to the model (Step 1). **c** Extraction of the high-contrast atomic columns forming the 12MR supercages (Step 2). **d** Acquisition of the *x*–*y* coordinates of each column (Step 3). **e** Addition of the center of the hexagonal feature (corresponding to the center of the 12MR supercage in MWW zeolite (Step 4). **f** Experimental iDPC image. **g** Extraction of the high-contrast atomic columns forming the 12MR supercages from the experimental iDPC image by template-matching method (Step 5). **h** Acquisition of the *x*–*y* coordinates of each column by fitting with 2D Gaussian function (Step 6). **i** Addition of the center of the hexagonal feature (corresponding to the center of the 12MR supercage in MWW zeolite) (Step 7). **j** Alignment of the experimental and theoretical features (i.e., the images in **e** and **i**) by minimizing the total atomic displacement (Step 8). This procedure provides the best fitting between pairs of points without any bias. Workflow for the strain analysis on the structural features extracted from the experimental iDPC images and the MWW zeolite model. **k** Delanuy triangulation on the experimental feature (Step 9). **l** Delanuy triangulation on the model (Step 10). Strain distribution in each triangle unit obtained by calculations on the experimental structural feature (Step 11). The pixel size in these images is 0.0174 nm.

The strain analysis of the Ir@MWW zeolite samples was performed using the information of the STEM-HAADF and STEM-iDPC paired images recorded along the [001] zone axis of MWW zeolite. In the experimental approach, STEM-HAADF images were used to determine the location of the Ir atoms/clusters, whereas STEM-iDPC images were processed to study the zeolite structure surrounding the Ir species. The workflow for the measurement of lattice strain in the MWW zeolite is illustrated in Fig. 2. In a first step, the *x–y* coordinates of the brightest atomic columns due to the proximity of Si atoms along the [001] direction, lying in projection on a hexagonal net, were extracted from the experimental iDPC image by template matching method (see Fig. 2a–c, Supplementary Note 1 and Supplementary Fig. 8 for further instruction). The *x–y* coordinates of each bright point were further refined by fitting to a 2D Gaussian function. As depicted in Fig. 2d, a set of points with sub-angstrom spatial precision laying on a net of edge-sharing hexagons is obtained for each STEM-iDPC image, which will be further used as markers to represent the structure of the measured MWW zeolite.

**Fig. 3:**
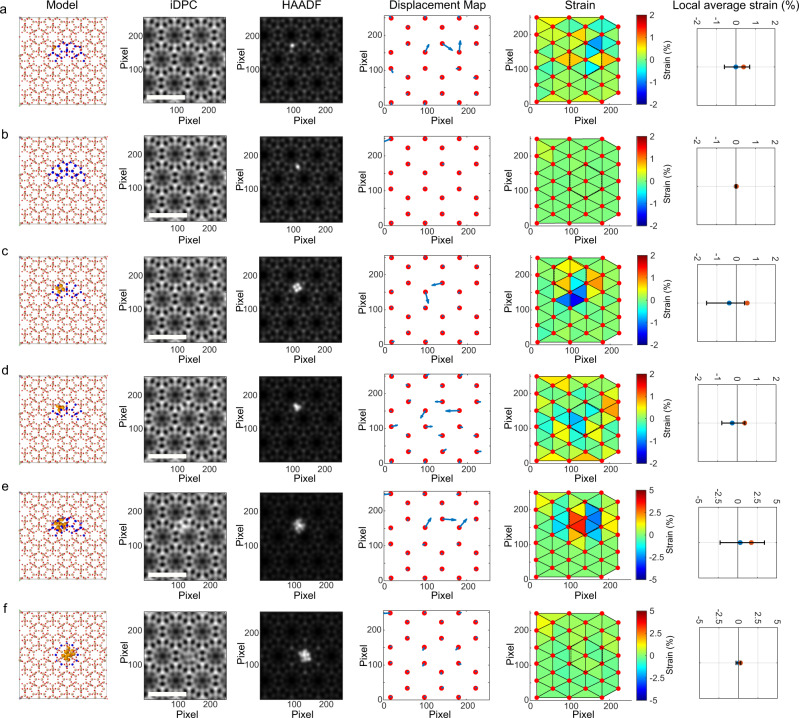
Simulation of the local strain in Ir@MWW models. Representative simulated iDPC images based on the geometry optimized DFT calculations and the local strain calculated according to the simulated iDPC images are shown in this figure. **a** Isolated Ir atom located in the 10MR window (Ir_1_–A structure), **b** isolated Ir atom at the 10MR window (Ir_1_–F structure), **c** Ir_4_ with a square planar geometry located at the 10MR channel (Ir_4_–J structure), **d** Ir_4_ with rhombic geometry located at the 10MR window between the supercages, (Ir_4_–M structure), **e** Ir_13_ cluster at the 10MR windows (Ir_13_–X structure) and **f** Ir_13_ cluster at the supercage (Ir_13_–T structure). The pixel size in these images is 0.0174 nm and the scale bar is 2 nm. In the right column, the strain range spanned by these triangles (segment bars) as well as the average of local strain values (blue dots) and the average of the absolute strain values (orange dot in the plots) are shown.

To describe and quantify the structural distortion in a target material, a reference in which no lattice distortion is expected should be employed for comparison. However, this assumption does not hold in the case of metal-zeolite materials prepared by hydrothermal synthesis, since intrinsic defects within the zeolite framework could be present at any location. Therefore, as will be shown later, an iDPC image simulated along the [001] zone axis, corresponding to a model of the pure-silica MWW zeolite refined by DFT calculations, is used as reference (see Fig. 3 and more details about the image simulation process in the “Methods” section). Once the iDPC simulated image is generated, the *x*–*y* coordinates of the hexagonal network of bright points, which are also associated to the Si atomic columns, were extracted as markers to represent the structure of the reference MWW zeolite.

To compare the positions of the two sets of *x–y* points (simulated and experimental), they were first aligned by minimizing the total atomic displacement (see Fig. 2i, j). This procedure provides bias-free fitting between pairs of points. Subsequently, as shown in Fig. 3a–d, both hexagonal nets were transformed into triangular ones by adding extra points at the centres of the hexagonal units through Delanuy triangulation and the area difference between the experimental and simulated reference structure is used as descriptor for the local strain (see Supplementary Fig. 9 and Supplementary Note 2 for further discussion on the Delanuy triangulation of the reference point network). Merging the experimental STEM-HAADF image with this strain map allows us to discriminate the influence of the Ir species on the local distortions of the zeolite framework. It is necessary to mention that the calculated strain at the edges of these maps can be eventually influenced by truncation of the contrasts at the borders.

### Strain analysis with simulated images

To validate the proposed methodology, representative Ir@MWW models optimized by DFT calculations (shown in Fig. 1, comprising subnanometric Ir species from isolated Ir atom to Ir_4_ and Ir_13_ clusters) have been employed to simulate the corresponding HAADF-STEM and iDPC-STEM images. Based on the DFT-optimized structures, several representative configurations were chosen to test the sensitivity of the methodology proposed for the measurement of local strain in the Ir@MWW materials, as presented in Fig. 3 and Supplementary Fig. 10. Although the contrasts of the metal species in the iDPC images are not as clear as those in HAADF images (due to the Z-dependent contrast in iDPC imaging mode), their impacts on the zeolite framework structure could be reflected through the displacements of the contrasts of the Si atomic columns, as discussed in Supplementary Note 3.

In all cases, the displacement field is high in the atomic columns close to the location of the Ir species. The distortion effects are rather local because the propagation of the local distortion is not observed at regions whose distance to Ir species are over 1.5 nm. As expected, the magnitude and direction of the displacements depends on the particular model.

A slightly positive strain (red/orange triangles) is detected in the vicinity of iridium in the Ir_1_ models where the metal, in projection, is located asymmetrically with respect to the reference network points (DFT structures Ir_1_–A, Ir_1_–C and Ir_1_–D). No strain is observed (green triangles) for the Ir_1_–F model, in which the Ir atom locates close to the center of the 10MR window.

To illustrate these results in quantitative terms, different strain figures were calculated considering the set of 6 triangles conforming the hexagon in the vicinity of the Ir species depicting the largest structural distortion, according to the displacement field maps. In particular, Fig. 3 and Supplementary Fig. 10 (right columns) plots both the strain range spanned by these triangles (segment bars) as well as the average of local strain values (blue dots). Note that in the Ir_1_ models, the average strain is close to zero, except for Ir_1_–D, which shows a net positive local strain. This is due to a compensation of positive and negative local strain values in these structures, while the Ir_1_–F structure show negligible strain in the whole map. To emphasize this difference, the average of the absolute strain values was also calculated (orange dot in the plots) to provide a clearer picture of the structural distortions in various models.

According to DFT calculations, in the Ir_1_–A, Ir_1_–C, and Ir_1_–D structures, breakage of the framework Si–O–Si bonds and formation of Ir–Si and Ir–O bonds leads to marked structural distortions in the Si and O atomic columns surrounding the Ir atom. Such distortions are translated into small lateral shifts, along the perpendicular direction of the MWW [001] zone axis. The subtle structural changes finally cause a slight shift of the center of the image contrasts corresponding to those columns (comprising both Si and O atoms). Consequently, some of the triangles, derived from the Delanuy triangulation processing of the extracted structural network, increase in area (shown in red), while the neighboring ones become smaller (shown in blue). In the Ir_1_–F structure, the weak interactions of Ir_1_ with the framework oxygen atoms do not cause marked shift of the Si columns, and thus, do not affect the displacement of the Si atomic column.

As displayed in Fig. 3c, d and Supplementary Fig. 10, Ir_4_ models give also rise to similar (Ir_4_–P) or even higher (Ir_4_–S) absolute average strain values in comparison with those observed for Ir_1_ structures. The local average strains are very close to zero in Ir_1_ structures, while a clearly negative average strain is observed for those models in which Ir_4_ bonds directly to framework oxygen atoms (Ir_4_–H and Ir_4_–R), though the absolute average strain in the whole map is close to that of Ir_1_–A structure.

As shown in Fig. 3e and Supplementary Fig. 10, the Ir_13_ models present local average strain values close to zero or slightly positive. Large values of the absolute local strain are observed when the Ir clusters locate at positions where the Si–O framework bonds are disrupted, particularly at the 10MR channel and at the 10MR window (Ir_13_–X and Ir_13_–W structures). In contrast, occupation of the cage by the Ir_13_ cluster (Ir_13_–T and Ir_13_–U structures) leads either to null or very moderate values of absolute local strain (see Fig. 3f and Supplementary Fig. 10).

**Fig. 4:**
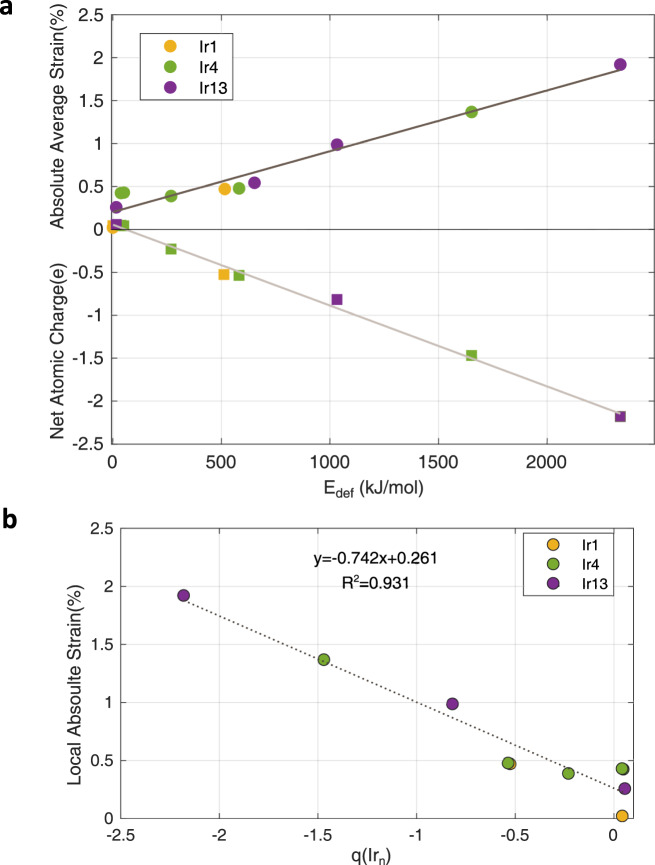
Correlation of the atomicity of Ir species to the local strain and net atomic charge. **a** In the positive y-axis, the values of the absolute local strain (circles) against the network deformation energy (E_def_) for all the simulated models are presented. The linear fitting (black line) displays a correlation value (R2) of 0.953. In the negative y-axis, net atomic charges of various Ir species (squares) versus deformation energy (E_def_) are shown. In this case, a R2 value of 0.992 is obtained after the linear fitting (light gray line). **b** Correlation of the local absolute strain measured in experimental images *versus* the net atomic charge of Ir species (qIr_n_) derived from theoretical calculations.

In an attempt to correlate the atomicity of the Ir species and the magnitude of strain, the values of the absolute local strain versus the network deformation energy (E_def_) for all the simulated models have been plotted in Fig. 4a (black line). In general, there is a reasonable linear fit between the two sets of data, particularly for those cases which involve significant structural distortions (i.e., E_def_ > 50 kJ/mol). Moreover, a comparison of this plot with that corresponding to the correlation between the cluster charge (qIr_n_) and deformation energy is also included in Fig. 4a (light gray line). The very similar tendency of the two fitting plots infers an interconnection between the electronic properties of Ir clusters and local strain surrounding the Ir clusters. In other words, the electronic and geometric features of the Ir species in confined environment can be correlated. The magnitude of structural distortion in zeolite framework does not depend primarily on the cluster size, but it is rather related to the electronic interactions between the Ir species and the zeolite framework.

The above analysis and results also show that the proposed methodology is sensitive to the subtle distortions within the structure of MWW zeolite, caused by the disruption of the Si–O–Si framework bonds as indicated by the theoretical calculations. Compressive and expansive strain regions are clearly detected in these cases, even when they are caused by an isolated Ir atom interacting with the framework.

Absolute strain appears as the parameter offering the adequate correlation with the intensity of the metal-zeolite interaction. By definition, this parameter takes into account the areal deviation of the triangle reference network. A modification of the area involves stretching/compressing the bonds in the zeolite structure. In principle, despite increasing or decreasing the area with respect to that in the perfect structure, the magnitude of its modification should be related somehow to the deformation energy involved. The good consistence between the theoretical and image simulation studies demonstrates the promise of the methodology for detecting confinement effects in practical metal-zeolite materials. Furthermore, it is demonstrated that such confinement involves concomitant electronic and steric components.

### Synthesis of Ir@MWW materials

To demonstrate the application of the strain analysis methodology to practical metal-zeolite materials, we have prepared a Ir@MWW-subnano sample comprising different types of subnanometric Ir species encapsulated in pure-silica MWW zeolite crystallites^28^. To avoid the beam-induced damage to the zeolite structures, we have carried out the image acquisition under low-dose conditions. The stability of the Ir@MWW-subnano sample has been tested by consecutively recording paired HAADF and iDPC images, which confirms the preservation of the zeolite structure in the first shot (see Supplementary Figs. 11–16 and related discussions in Supplementary Note 4–5).

As shown in Supplementary Figs. 17–18, the majority of the Ir species are Ir clusters of 0.5–0.7 nm while a small percentage of isolated Ir atoms and very small Ir clusters are also found in it. Considering the low Ir loading, the imaging depth of the working conditions of STEM-iDPC measurements and the morphology of MWW zeolite crystallites (see Supplementary Fig. 19), the particles with bright contrast in the HAADF-STEM images are considered as individual species instead of overlapping image of several Ir particles along the [001] direction (see discussion in Supplementary Note 6).

As shown in our previous work, the location of the subnanometric Ir clusters can be determined by the combination of HAADF and iDPC images and these Ir species are mostly located at or near to the 10MR windows. The regioselective generation of Ir species observed in the synthesized materials is well consistent with the theoretical calculation results because the Ir species, especially the Ir_4_ clusters, can be well accommodated and stabilized by the 10MR windows.

Herein, we would like to discuss several features observed in the prior experimental work that can be further elaborated by the DFT calculation results shown in this present work. The isolated Ir atoms are located at the region of the 10MR windows according to the STEM-iDPC imaging technique, though their exact location cannot be directly determined. Inferred by the calculation results, it is highly possible that the Ir atoms are stabilized by the 5MR unit of the 10MR window. Indeed, as displayed in Supplementary Fig. 20, we have observed an area comprising an isolated Ir atom in good match with the simulated images of Ir_1_–C structure. Moreover, the Ir–O bonding between Ir species and zeolite framework suggested by DFT calculations are also observed by extended X-ray absorption fine structure (EXAFS) spectra (see Supplementary Fig. 21). However, the Ir–Si bonding suggested by the DFT calculations is not observed because of the low percentage of isolated Ir atoms in the Ir@MWW-subnano sample. The cross validation between the theoretical and experimental results indicates the rationality of the proposed models and methodology. Nevertheless, these further insights show the importance of combining atomic-level structural characterizations and theoretical studies for elucidating the detailed structures of subnanometric metal species confined in a porous matrix.

### Strain analysis in Ir@MWW material

Thanks to the presence of several types of Ir species in a single Ir@MWW zeolite sample (see STEM-HAADF images in Supplementary Figs. 9–10), such sample can be used to test the size-dependent local strain caused by metal-zeolite interaction, because the STEM-iDPC measurements can be performed under almost identical conditions to minimize the influence of the experimental set-up. Nevertheless, HAADF and iDPC images were acquired under low-dose conditions to avoid beam-induced modifications to the Ir@MWW sample^23^.

Firstly, areas where no Ir species are measured as internal reference and this approach can avoid the influence of subtle changes in the actual magnification values between different samples, due to small differences in the excitation conditions of the lens system. According to the results presented in Supplementary Fig. 22, the mean values of the two strain parameters is −0.6% for the local net strain and +1.9% for the absolute local strain and these values will be taken as an offset in the analysis of strain maps containing Ir species and, therefore, will be subtracted from the values determined in these areas.

**Fig. 5:**
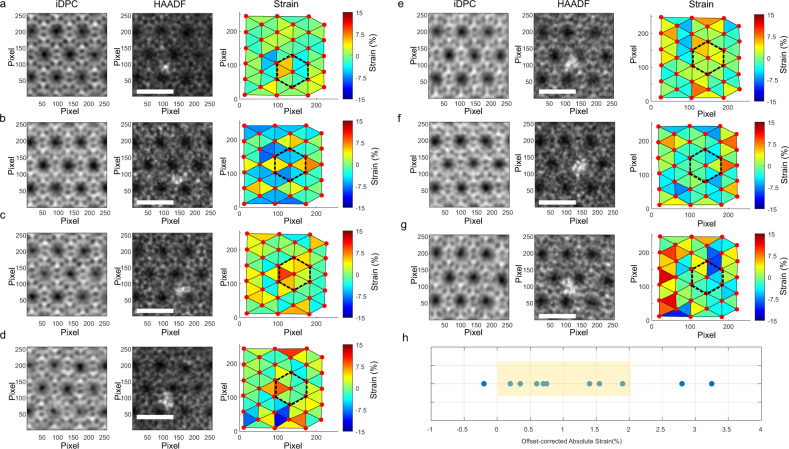
Experimental measurements of the local strain in Ir@MWW-subnano sample. The paired HAADF and iDPC images of multiple areas with different types of Ir species have been measured. **a**, **b** Areas with an isolated Ir atom, **c**–**g** Areas with Ir clusters located close to the 10MR window. **h** Offset-corrected absolute local strain obtained in different areas. The pixel size in these images is 0.0174 nm and the scale bar is 2 nm.

Different areas of HAADF-iDPC paired images, where both isolated atoms (Fig. 5a, b) and subnanometric Ir clusters (Fig. 5c–g) were detected, are summarized in Fig. 5 and Supplementary Fig. 23. Triangles showing stretching (orange-red ones) and compression (blue ones) are found surrounding the Ir species, suggesting the structural changes of the zeolite framework. After considering the offset-corrected absolute strain, the values determined from the whole set of HAADF-iDPC image pairs are presented in Supplementary Table 1. Importantly, as shown in Fig. 5h, most experimental images (~75%) give rise to results within the range (0–2%) determined from the image simulation results based on the models derived from DFT calculations. Half the analyzed images provide absolute strain values in the 0.2–0.7% range, corresponding to Ir clusters with moderate interaction (E_def_ < 700 kJ/mol) with the zeolite framework. This is consistent with the size distribution of the Ir clusters in the Ir@MWW-subnano sample because the sizes of most encapsulated Ir clusters are ~0.6 nm, which can be considered as intermediates between the Ir_4_ and Ir_13_ structures built in Fig. 1.

Furthermore, we have also observed a linear correlation between the absolute local strain and electronic properties of the confined Ir species (see Fig. 4b), confirming the effectiveness of the image analysis approach for detecting the local structural distortion in experimental images and its high precision. The theoretical calculation results indicate that, the structural distortion caused by the Ir-zeolite interaction may be related to the location of the Ir species in the zeolite structure. Indeed, the impacts of the location can be reflected in the site-dependent local strain observed with the large Ir clusters at 10MR window and the 12MR supercage (see Supplementary Fig. 24 and Supplementary Note 7). Though some errors and limitations associated with the methodology and the defects in the Ir@MWW sample need to be noted (see the related discussions in Supplementary Note 8), the above results are still very promising and encouraging, when considering the difficulty for measuring the elusive confinement effects in metal-zeolite materials.

The host-guest interaction within zeolite structure has previously been investigated experimentally by electronic and vibrational spectroscopic techniques^29–31^. Formation of cavity-dependent transition states or intermediates inside zeolite structures has also been observed and then associated with the catalytic properties^32,33^. From an electronic point of view, the abovementioned host-guest interaction between encapsulated molecules is realized through van der Waals forces^34^, while the interaction between metal-zeolite framework is realized through ionic and/or coordination interaction via electron transfer between the metal cluster and oxygen atoms in zeolite framework. Different to the previous works showing the confinement effect within zeolite structure in a global and averaged manner, the results shown in this work are directly associated to the active sites, i.e. subnanometric Ir clusters, and the surrounding zeolite framework. The relationship observed between the local absolute strain in zeolite framework and the electronic features of metal species in both experimental and theoretical studies can provide insights for a better understanding of the metal-molecule interaction in constrained environment^35,36^.

In principle, the concept and methodology demonstrated in this work could also be adapted to other porous materials with the encapsulation of metal species, such as metal-organic frameworks^37^. Measuring the local structures of the metal sites can provide insights to understand the coordination environment of isolated metal atoms and clusters. Specially, measurements performed under in situ conditions, on the basis of proper experimental design to avoid beam-induced damage, employment of advanced hardware (high-performance electron STEM detectors and CMOS cameras) and data analysis methods (artificial intelligence-assisted imaging processing and analysis), would allow further insights on metal-molecule interactions, a key to better rationalize the catalytic properties^38^.

## Methods

### Computational details

Periodic density functional calculations were performed at the PBE^39^ level of theory with dispersion corrections included using the D3 Grimme’s method^40^ as implemented in the VASP code^41,42^. The valence density was expanded in a plane wave basis set with a kinetic energy cut-off of 600 eV, and the effect of the core electrons in the valence density was taken into account by means of the projector augmented wave (PAW) formalism^43^. Integration in the reciprocal space was carried out at the Γ k-point of the Brillouin zone. All calculations are spin-polarized, and the magnetic moment was allowed to evolve during the geometry optimizations. Electronic energies were converged to 10^−6^ eV and geometries were optimized until forces on atoms were less than 0.01 eV/Å Net atomic charges were calculated using the charge localization scheme of Bader^44,45^.

MWW crystallizes in a hexagonal P6/mmm space group with lattice parameters a = b = 14.390 Å and c = 25.198 Å, and contains 216 atoms in the conventional unit cell (72 Si and 144 O). The MWW silicon framework is composed of four-, five- and six-membered rings that link to form two independent channel systems, a bi-dimensional sinusoidal 10MR channel system (4.1 × 5.1 Å) running within the layers, and a second one formed by 12MR supercages (7.1 × 7.1 × 18.2 Å) interconnected by 10MR windows. In a first step, the atomic positions and unit cell parameters of the pure silica MWW framework were optimized without restrictions at the PBE-D3 level. The values obtained, a = b = 14.450 Å and c = 25.196 Å, show excellent agreement with the experimental ones and were used in all subsequent geometry optimizations. Then, Ir single atoms (Ir_1_), small Ir clusters with four Ir atoms (Ir_4_) and larger Ir clusters with thirteen atoms (Ir_13_) were placed at different positions within the MWW zeolite structure and the geometry of the MWW-Ir_n_ systems was fully optimized without restrictions for the atomic positions, keeping the volume and shape of the unit cell fixed at the PBE-D3 optimized values.

For each Ir_n_ atomicity, the relative stability of the different locations was evaluated through the relative energy E_rel_ with respect to the most stable position:

1 $${{{{{{\rm{E}}}}}}}_{{{{{{\rm{rel}}}}}}}={{{{{{\rm{E}}}}}}}_{{{{{{\rm{Irn}}}}}}@{{{{{\rm{MWW}}}}}}}{-}{({{{{{{\rm{E}}}}}}}_{{{{{{\rm{Irn}}}}}}@{{{{{\rm{MWW}}}}}}})}_{{{{{{\rm{most}}}}}}{{{{{\rm{stable}}}}}}}$$

The stabilization of the Ir_n_ atoms and clusters by interaction with the zeolite framework was evaluated through the encapsulation energy E_enc_ calculated according to:

2 $${{{{{{\rm{E}}}}}}}_{{{{{{\rm{enc}}}}}}}={{{{{{\rm{E}}}}}}}_{{{{{{\rm{Irn}}}}}}@{{{{{\rm{MWW}}}}}}}{-}{{{{{{\rm{E}}}}}}}_{{{{{{\rm{MWW}}}}}}}{-}{{{{{{\rm{E}}}}}}}_{{{{{{\rm{Irn}}}}}}}$$

where E_Irn@MWW_ is the total energy of the system with Ir_n_ incorporated in the zeolite, E_MWW_ is the total energy of the optimized pure silica MWW framework and E_Irn_ is the energy of an Ir_1_ atom or an Ir_n_ cluster in vacuum in its most stable electronic state.

Finally, to evaluate the strain suffered by the zeolite framework upon incorporation of Ir_n_ atoms or clusters, the deformation energy E_def_ was calculated as:

3 $${{{{{{\rm{E}}}}}}}_{{{{{{\rm{def}}}}}}}={{{{{{\rm{E}}}}}}}_{{{{{{\rm{MWW}}}}}}({{{{{\rm{Irn}}}}}})}{-}{{{{{{\rm{E}}}}}}}_{{{{{{\rm{MWW}}}}}}}$$

E_MWW(Irn)_ is the total energy of the pure silica zeolite framework without Ir atoms, obtained from a single point calculation in which the Si and O atoms are fixed at their optimized positions in the Ir_n_-MWW system. In all cases, the pure-silica systems without Ir converged correspond to a singlet state.

### Simulation of HAADF-iDPC STEM image pairs

STEM image simulation was carried out using TEMSIM software^46^. The complex structural models used as input in these simulations were built using the Rhodius software developed at Universidad de Cadiz^47^. It is important to mention that the model employed in the simulation was scaled to the experimental image using the pixel size of the latter. iDPC images map the projected potential of the atomic columns, however, in order to evaluate the intrinsic error associated to the procedure employed to extract the x–y coordinates of the hexagonal net, the coordinates obtained from the iDPC image simulation were compared with those corresponding to the structural model.

Specifically, the TEMSIM software was used to simulate both the HAADF-STEM image and the images of each quadrant of the segmented DF detector, using the optoelectronic parameters described below: HT = 300 kV, Cs3 = 0.001 mm, Cs5 = 5 mm, Δf = −3 nm, convergence angle = 18 mrad, HAADF detector size = 42-198 mrad. The segmented dark-field detector size for each quadrant: Q1 = 12 42 seg 0 90; Q2 = 12 42 seg 90 180; Q3 = 12 42 seg -180 -90; Q4 = 12 42 seg -90 0. The orientation of each quadrant corresponds to the FEI Titan Themis3 60-300 microscope, in which the STEM experiments were performed.

Once each of the 4 segment DF images were calculated, the DPC and iDPC images were calculated following the equations (46) and (47) described in the literature, respectively, using a home-made script in Digital Micrograph^25^. A convolution with a Gaussian low pass filter was finally applied to both the iDPC and HAADF images to remove details beyond the resolution of experimental images.

### Synthesis of Ir@MWW sample

The Ir@MWW-subnano sample was prepared via a one-pot synthesis strategy, which allows the incorporation of subnanometric Ir species in MWW zeolite crystallites. In a typical procedure, 0.237 g of NaCl was dissolved in a mixture of 6.64 g of N,N,N-trimethyl-1-adamantanamonium hydroxide solution (0.8 mol/L) and 5.0 g distilled water. Then, 0.700 g of hexamethyleneimine, 1.0 g of IrCl_3_∙xH_2_O (supplied by Sigma-Aldrich, product code: 206245-1 G) solution prepared by 50 mg of IrCl_3_∙xH_2_O and 5 g of H_2_O, 150 μL of ethylenediamine was added to this solution. The above solution was kept stirring at room temperature for 2 h. Then, 1.22 g of fumed silica (Aerosil 200, Degussa) was added under continuous stirring. After 3 h, the resultant suspension was transferred to a Teflon-lined stainless-steel autoclave and then heated at 150 °C for 120 h under agitation conditions (60 rpm). After the hydrothermal process, the solid product was isolated by filtration and washed with distilled water and acetone and then dried at 60 ^o^C. Then the solid sample was calcined in flow air at 560 °C with a ramp rate of 2 °C/min and then kept at 560 °C for 8 h. After calcination in air, the solid material was reduced by H_2_ at 650 °C, resulting in the formation of Ir@MWW-subnano material comprising subnanometric Ir speices.

### Characterization by TEM

Samples for electron microscopy studies were prepared by dispersing the solid sample on holey-carbon coated copper grids using CH_2_Cl_2_ as the solvent. Electron Microscopy measurements were performed using two types of microscopes. A non-corrected JEOL 2100F microscope working at 200 kV was used to record High Angle Annular Dark-Field (HAADF) images at low resolution to check the dispersion of Ir species. High-resolution HAADF-STEM and STEM-iDPC images were recorded on a FEI Titan3 Themis 60-300 microscope working at 300 kV, which is equipped with double aberration correctors and a monochromated electron beam source. iDPC (Integrated-Differential Phase Contrast) imaging, in which the contrasts are related to the atomic number of the elements in the material, is performed with the 4-segment detector equipped within the microscope. The iDPC imaging technique allows imaging light elements such as O (Z = 8) in the presence of heavier ones (such as Si, Z = 14) under low-dose conditions. For each image recorded, HAADF-iDPC paired images with a size of 2048 × 2048 pixels were recorded simultaneously using a convergence angle of 18.6 mrad and a camera length of 91 mm. In order to minimize the beam damage to zeolite structure, the images were recorded with a beam current of ~10 pA, a 0.625–1.25 μs dwell time and an automated fine-tuning alignment of A1 and C1 using the OptiSTEM software. An image processing methodology for the analysis of the experimental HAADF-iDPC images has been developed improve the signal/noise ratio. First, the HR-HAADF STEM images were denoised by combining the Anscomb transform and Undecimated Wavelet Transforms (UWVT) treatments, which are coded within a Matlab script.

## Supplementary information

Supplementary information

## Data Availability

The experimental data that support the findings of this study are available from the corresponding author upon request.

## Code availability

The code and scripts used in this work are available from the corresponding author upon request.

## References

[1] Grommet AB, Feller M, Klajn R (2020). Chemical reactivity under nanoconfinement. Nat. Nanotechnol..

[2] Fu Q, Yang F, Bao X (2013). Interface-confined oxide nanostructures for catalytic oxidation reactions. Acc. Chem. Res..

[3] Gounder R, Iglesia E (2012). The roles of entropy and enthalpy in stabilizing ion-pairs at transition states in zeolite acid catalysis. Acc. Chem. Res..

[4] Sastre G, Corma A (2009). The confinement effect in zeolites. J. Mol. Catal. A: Chem..

[5] Boronat M, Corma A (2019). What is measured when measuring acidity in zeolites with probe molecules?. ACS Catal..

[6] Li C (2011). Chiral synthesis on catalysts immobilized in microporous and mesoporous materials. Catal. Rev..

[7] Thomas JM, Raja R (2008). Exploiting nanospace for asymmetric catalysis: confinement of immobilized, single-site chiral catalysts enhances enantioselectivity. Acc. Chem. Res..

[8] Cho HJ (2019). Molecular-level proximity of metal and acid sites in zeolite-encapsulated Pt nanoparticles for selective multistep tandem catalysis. ACS Catal..

[9] Wang N, Sun Q, Yu J (2019). Ultrasmall metal nanoparticles confined within crystalline nanoporous materials: a fascinating class of nanocatalysts. Adv. Mater..

[10] Wu SM, Yang XY, Janiak C (2019). Confinement effects in zeolite-confined noble metals. Angew. Chem. Int. Ed..

[11] Wang L, Xu S, He S, Xiao F-S (2018). Rational construction of metal nanoparticles fixed in zeolite crystals as highly efficient heterogeneous catalysts. Nano Today.

[12] Liu L, Corma A (2020). Confining isolated atoms and clusters in crystalline porous materials for catalysis. Nat. Rev. Mater..

[13] Liu L (2017). Generation of subnanometric platinum with high stability during transformation of a 2D zeolite into 3D. Nat. Mater..

[14] Liu L (2019). Regioselective generation and reactivity control of subnanometric platinum clusters in zeolites for high-temperature catalysis. Nat. Mater..

[15] Aydin C (2011). Tracking iridium atoms with electron microscopy: first steps of metal nanocluster formation in one-dimensional zeolite channels. Nano Lett..

[16] Warner JH, Young NP, Kirkland AI, Briggs GA (2011). Resolving strain in carbon nanotubes at the atomic level. Nat. Mater..

[17] Mukherjee D, Gamler JTL, Skrabalak SE, Unocic RR (2020). Lattice strain measurement of Core@Shell electrocatalysts with 4D scanning transmission electron microscopy nanobeam electron diffraction. ACS Catal..

[18] Oh MH (2020). Design and synthesis of multigrain nanocrystals via geometric misfit strain. Nature.

[19] Lopez-Haro M (2016). Strain field in ultrasmall gold nanoparticles supported on cerium-based mixed oxides. key influence of the support redox state. Langmuir.

[20] You B (2019). Enhancing electrocatalytic water splitting by strain engineering. Adv. Mater..

[21] Yang S, Liu F, Wu C, Yang S (2016). Tuning surface properties of low dimensional materials via strain engineering. Small.

[22] Wu J (2012). Surface lattice-engineered bimetallic nanoparticles and their catalytic properties. Chem. Soc. Rev..

[23] Ortalan V, Uzun A, Gates BC, Browning ND (2010). Direct imaging of single metal atoms and clusters in the pores of dealuminated HY zeolite. Nat. Nanotechnol..

[24] Juneau M (2020). Characterization of metal‐zeolite composite catalysts: determining the environment of the active phase. ChemCatChem.

[25] Lazic I, Bosch EGT, Lazar S (2016). Phase contrast STEM for thin samples: integrated differential phase contrast. Ultramicroscopy.

[26] Leonowicz ME, Lawton JA, Lawton SL, Rubin MK (1994). MCM-22: a molecular sieve with two independent multidimensional channel systems. Science.

[27] Hou D, Grajciar L, Nachtigall P, Heard CJ (2020). Origin of the unusual stability of zeolite-encapsulated sub-nanometer platinum. ACS Catal..

[28] Liu L (2020). Regioselective generation of single-site iridium atoms and their evolution into stabilized subnanometric iridium clusters in MWW zeolite. Angew. Chem. Int. Ed..

[29] Márquez F, García H, Palomares E, Fernández L, Corma A (2000). Spectroscopic evidence in support of the molecular orbital confinement concept: case of anthracene incorporated in zeolites. J. Am. Chem. Soc..

[30] Gallego EM (2017). “Ab initio” synthesis of zeolites for preestablished catalytic reactions. Science.

[31] Li C (2018). Synthesis of reaction‐adapted zeolites as methanol-to-olefins catalysts with mimics of reaction intermediates as organic structure‐directing agents. Nat. Catal..

[32] Li J (2014). Cavity controls the selectivity: insights of confinement effects on MTO reaction. ACS Catal..

[33] Goetze J, Yarulina I, Gascon J, Kapteijn F, Weckhuysen BM (2018). Revealing lattice expansion of small-pore zeolite catalysts during the methanol-to-olefins process using combined operando X-ray diffraction and UV-vis spectroscopy. ACS Catal..

[34] Shen B (2021). A single-molecule van der Waals compass. Nature.

[35] Vogiatzis, K. D., Li, G., Hensen, E. J. M., Gagliardi, L. & Pidko, E. A. Electronic structure of the [Cu_3_(u-O)_3_]^2+^ Cluster in Mordenite Zeolite and Its Effects on the Methane to Methanol Oxidation. *J. Phys. Chem. C* **121**, 22295–22302 (2017). 10.1021/acs.jpcc.7b08714 PMC5641944 29051794

[36] Harris JW, Bates JS, Bukowski BC, Greeley J, Gounder R (2020). Opportunities in catalysis over metal-zeotypes enabled by descriptions of active centers beyond their binding site. ACS Catal..

[37] Liu L (2019). Imaging defects and their evolution in a metal-organic framework at sub-unit-cell resolution. Nat. Chem..

[38] Liu L (2018). Evolution and stabilization of subnanometric metal species in confined space by in situ TEM. Nat. Commun..

[39] Perdew JP, Burke K, Ernzerhof M (1996). Generalized gradient approximation made simple. Phys. Rev. Lett..

[40] Grimme S, Antony J, Ehrlich S, Krieg S (2010). A Consistent and accurate ab initio parametrization of density functional dispersion correction (DFT-D) for the 94 elements H-Pu. J. Chem. Phys..

[41] Hafner J (2008). Ab-initio simulations of materials using VASP: density-functional theory and beyond. J. Comput. Chem..

[42] Kresse G, Furthmüller J (1996). Efficient iterative schemes for Ab initio total-energy calculations using a plane-wave basis set. Phys. Rev. B.

[43] Blöchl PE (1994). Projector augmented-wave method. Phys. Rev. B.

[44] Henkelman G, Arnaldsson A, Jónsson H (2006). A fast and robust algorithm for Bader decomposition of charge density. Comput. Mater. Sci..

[45] Sanville E, Kenny SD, Smith R, Henkelman G (2007). Improved grid-based algorithm for Bader charge allocation. J. Comp. Chem..

[46] López-Haro M (2018). A macroscopically relevant 3D-metrology approach for nanocatalysis research. Part. Part. Syst. Charact..

[47] Kirkland, E. J. *Advanced Computing in Electron Microscopy* (Springer, 2010).

