# Supplementary material for: Direct assessment of confinement effect in zeolite-encapsulated subnanometric metal species
Source: Nat Commun. 2022 Feb 10;13:821. doi: 10.1038/s41467-022-28356-y (PMC8831493; doi:10.1038/s41467-022-28356-y)
Supplement: Supplementary file 1 — Supplementary information [file 41467_2022_28356_MOESM1_ESM.pdf]

Supplementary Information

Direct assessment of confinement effect in zeolite-encapsulated subnanometric metal species

Lichen Liu[1,2]*, Miguel Lopez-Haro[3], Jose Antonio Perez-Omil[3], Mercedes Boronat[1], Jose J. Calvino[3] and Avelino Corma[1]*

[1] *Instituto de Tecnología Química, Universitat Politècnica de València-Consejo Superior de Investigaciones Científicas, Av. de los Naranjos s/n, Valencia 46022, Spain*

[2] *Department of Chemistry, Tsinghua University, Beijing 100084, China*

[3] *Departamento de Ciencia de los Materiales e Ingeniería Metalúrgica y Química Inorgánica, Facultad de Ciencias, Universidad de Cádiz, Cádiz, Spain*

*Corresponding author. Email: lichenliu@mail.tsinghua.edu.cn (L.L.) acorma@itq.upv.es (A.C.)

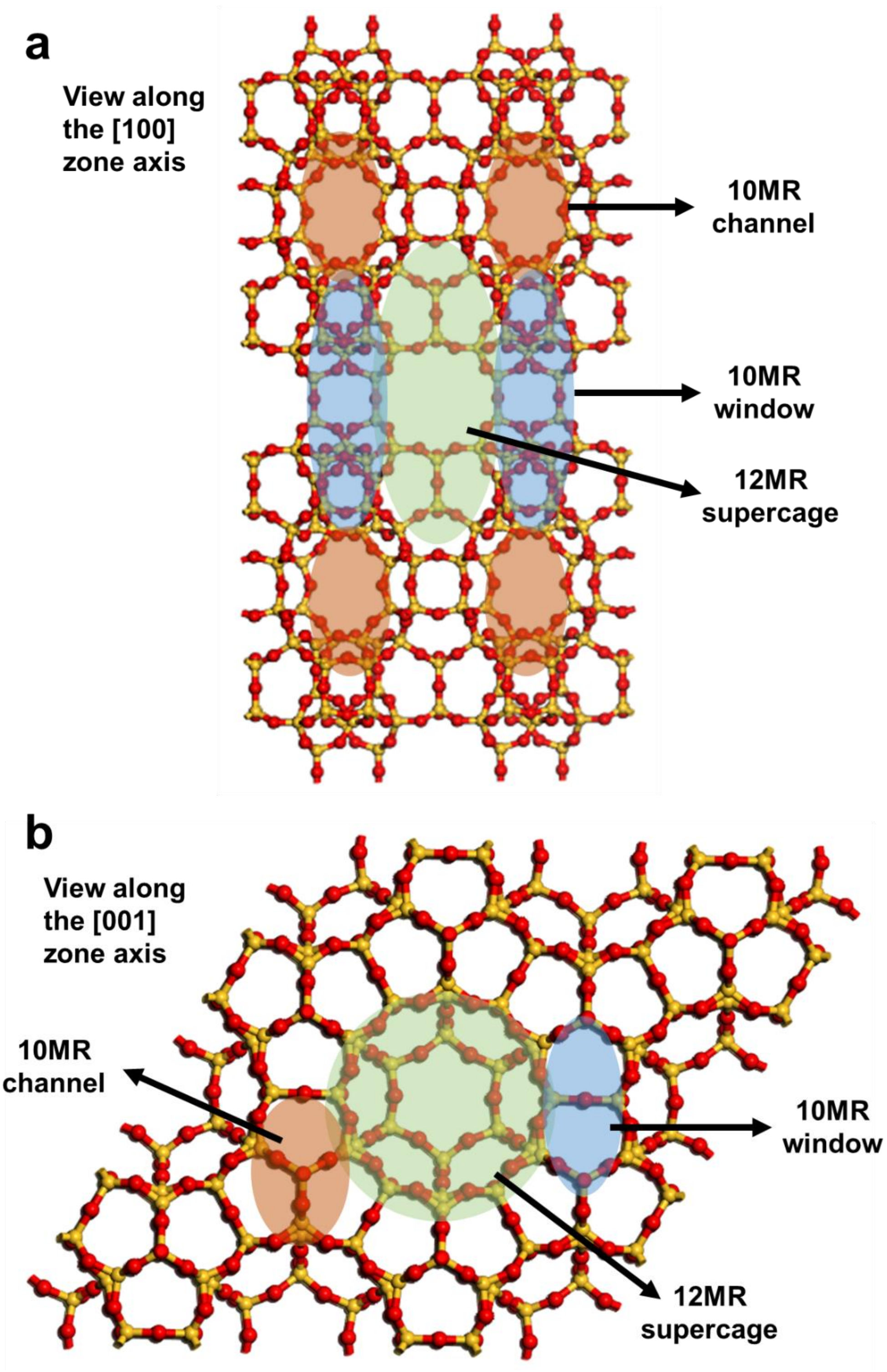

Supplementary Figure 1. Structural features of MWW zeolite. Schematic illustration of the three types of cavities/channels along the [100] (shown in a) and along the [001] (shown in b) zone axis. More information of MWW zeolite can be referred to the Database of Zeolite Structure in the website of International Zeolite Association. (https://asia.iza-structure.org/IZA-SC/framework.php?STC=MWW)

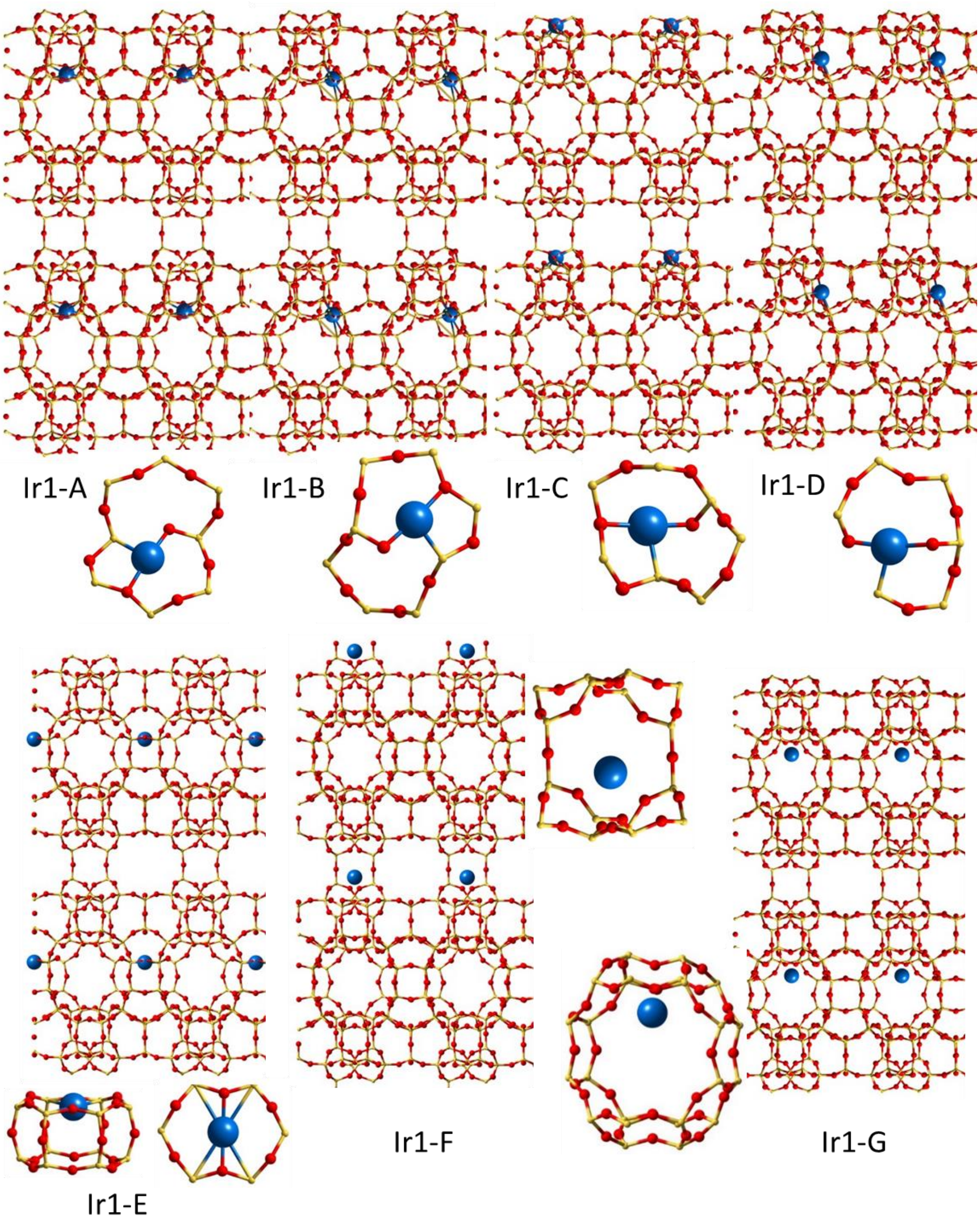

Supplementary Figure 2. Optimized geometries of Ir_1 atoms in different locations of the pure silica MWW zeolite.

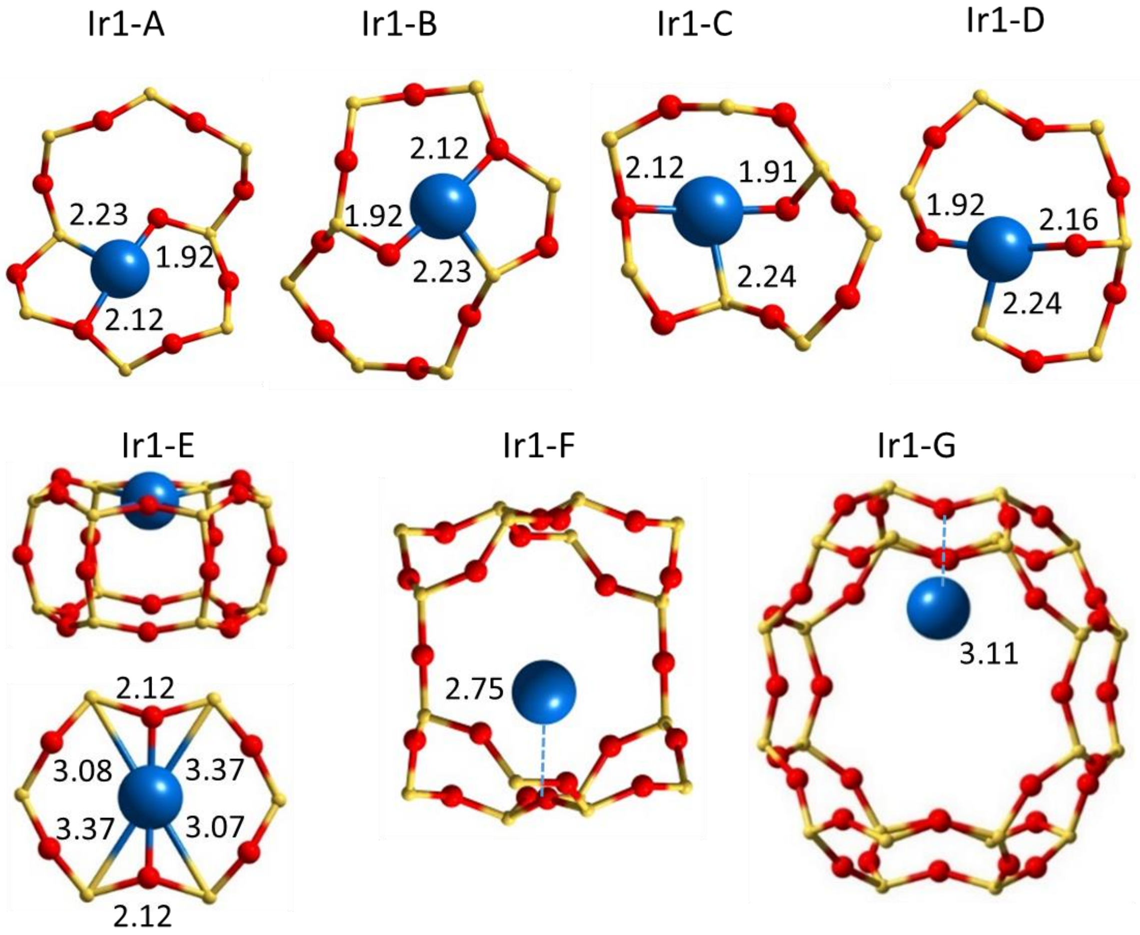

Supplementary Figure 3. Local geometry around isolated Ir atoms in different locations of the pure-silica MWW zeolite. The PBE-D3 optimized Ir-Si and Ir-O distances are also given in these models (in Å).

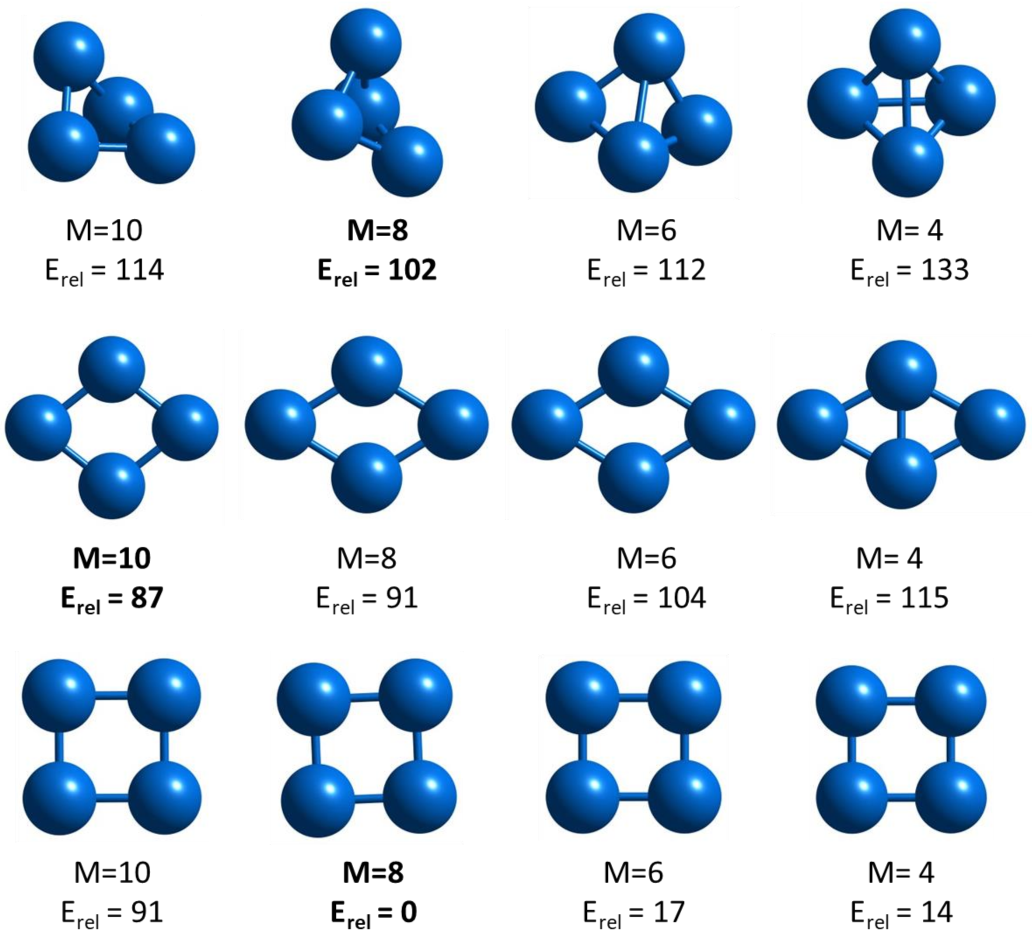

Supplementary Figure 4. Optimized geometry of different isomers of Ir4 clusters in vacuum. The total magnetization M (number of unpaired electrons) and the relative stability with respect to the global minimum (E_{rel} in kJ/mol) are given for each structure.

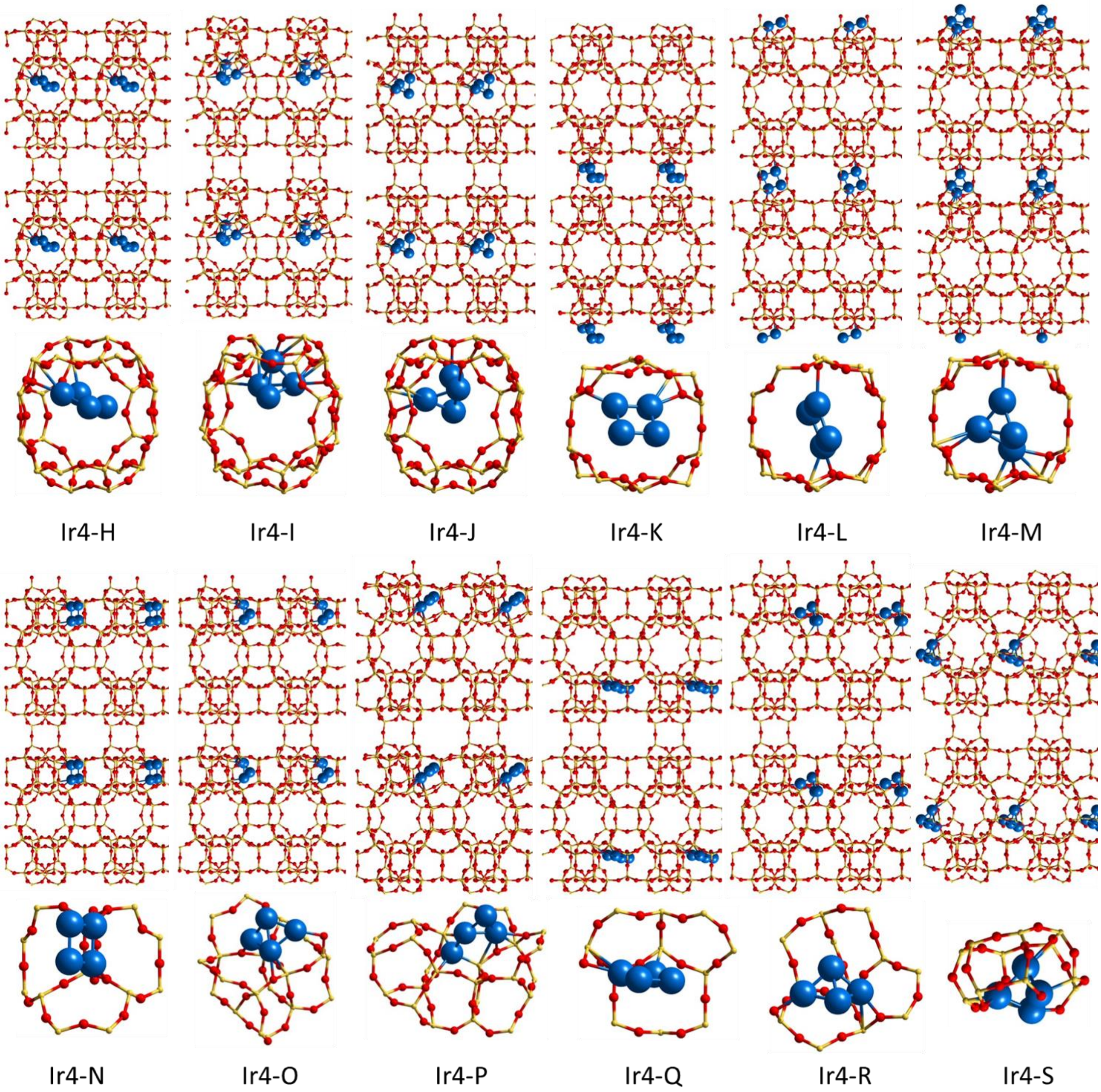

Supplementary Figure 5. Optimized geometries of Ir_4 clusters in different locations of the pure silica MWW zeolite.

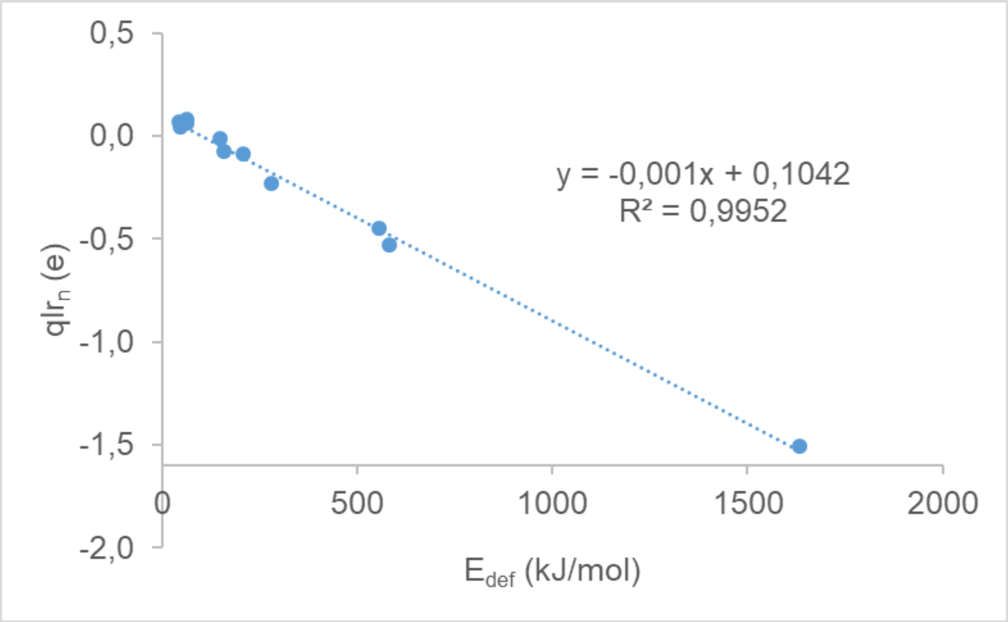

Supplementary Figure 6. Correlation between the charge transferred to the Ir_4 clusters and the deformation energy associated to the strain of the MWW zeolite framework.

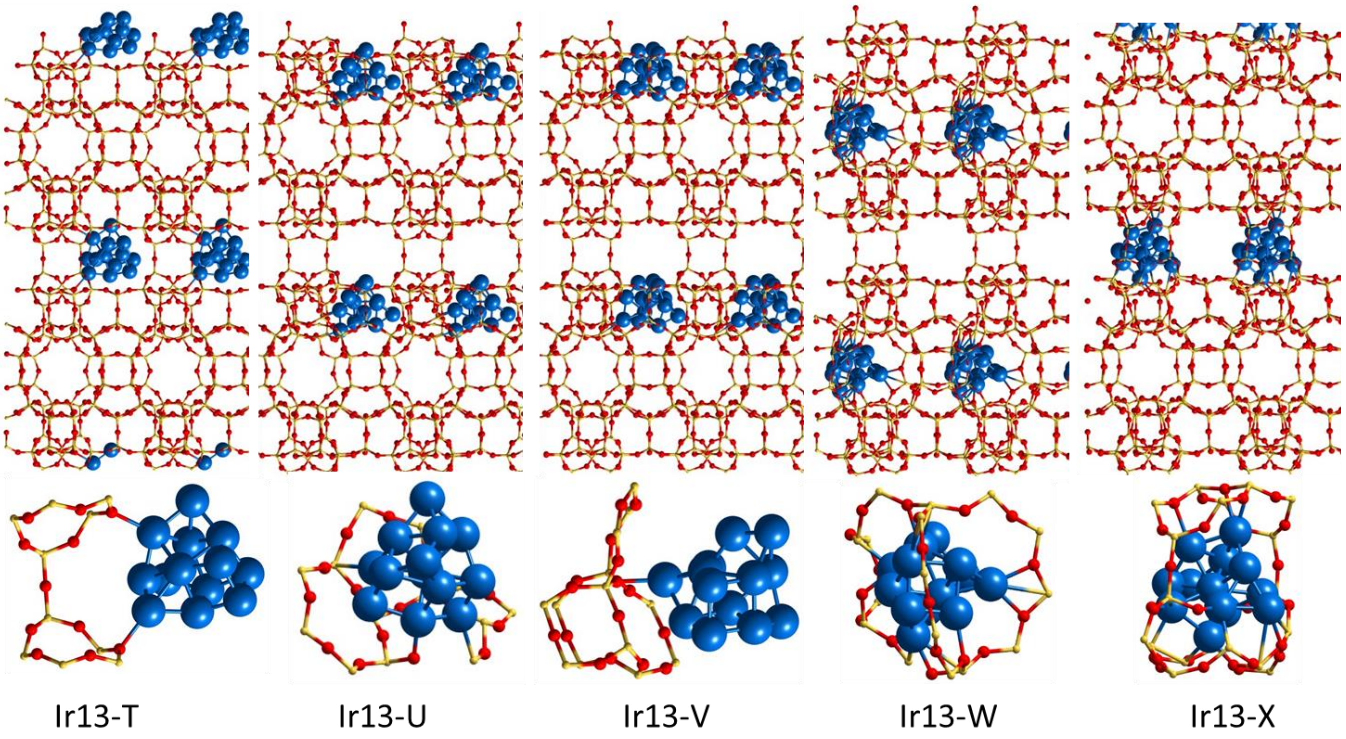

Supplementary Figure 7. Optimized geometries of Ir_{13} clusters in different locations of pure-silica MWW zeolite.

Supplementary Note 1

In principle, the local structural distortion is better to evaluated by directly using the positions of the Si atomic columns in the zeolite framework. However, as discussed before in the answers to the Reviewer and also in our manuscript, the vulnerable nature of zeolite framework under e-beam does not allow us to acquire high-quality image to determine the loation of single Si atoms in either the HAADF-STEM or the iDPC-STEM images, because of the very low contrast of the Si atoms under our imaging conditions.

It should be noted that, the reference points extracted from the iDPC images do not correspond to the Si atoms in the 12MR. Instead, the “bright point” in the iDPC images correspond to the centre of several 5MR units, as marked in **Supplementary Figure 8**. The high contrast of this specific location could be associated with its high charge density, as confirmed by the simulated iDPC image. The contrast of these structural features (6 points in a 12MR supercage) allows us to use them as the reference points to evaluate the local structural changes by methodology developed in this work, because the variation of the structures of zeolite framework will be reflected in the position of these reference points, as shown in the image simulation and DFT calculation results.

Regarding the four points with relatively weak contrast appeared **Figure 2** in the manuscript, they should be associated with the Si atoms at the 10MR window connecting the 12MR supercages, as illustrated in **Supplementary Figure 8**. Because the contrast of the four points is not high enough to provide reliable and precise spatial information to represent the structural feature of the zeolite framework, they are not extracted from the images for strain analysis.

To localize and extract the x-y coordinates of the atomic columns, an automatic approach based on template matching method was used. In particular, the identification was performed by using normalized cross correlation (NCC) with a Gaussian template.[1] NCC is a robust algorithm that allows to measure image similarity, *i.e.* this correlation method determines how well the template fits in each of the image pixels. The results provided by NCC can be displayed as an image in which the values of image similarity at each pixel are represented in an intensity scale where +1 and -1 correspond to maximal and minimal correlation, respectively.

Then, the NCC image has to be “filtered” using a threshold value, in the 0 to 1 range, which fixes the minimum correlation admitted as an acceptable match between the template and the corresponding atomic column. In other words, thresholding allows selecting those positions depicting the highest similarity between the template and the atomic columns. A threshold close to 1 demands a very high similarity cross-correlation whereas a low threshold, close to 0, includes locations where a low cross-correlation is detected.

As written in the original manuscript, to study local strain we focused on the 12MR supercages atomic columns since: (1) these are the ones showing the highest contrasts in the iDPC images and, therefore, those detected with highest reliability; (2) these are the positions in where the Ir clusters are mostly located.

The images displayed in **Figures 2c** and **Figure 2g** show the atomic columns automatically detected with a threshold of 0.7. Note that this value provides a reasonable identification of the highest contrast atomic columns but also picks up some atomic columns which actually belong to the 5 MR. To correct for this misidentification, the x-y coordinates of the latter were manually removed.

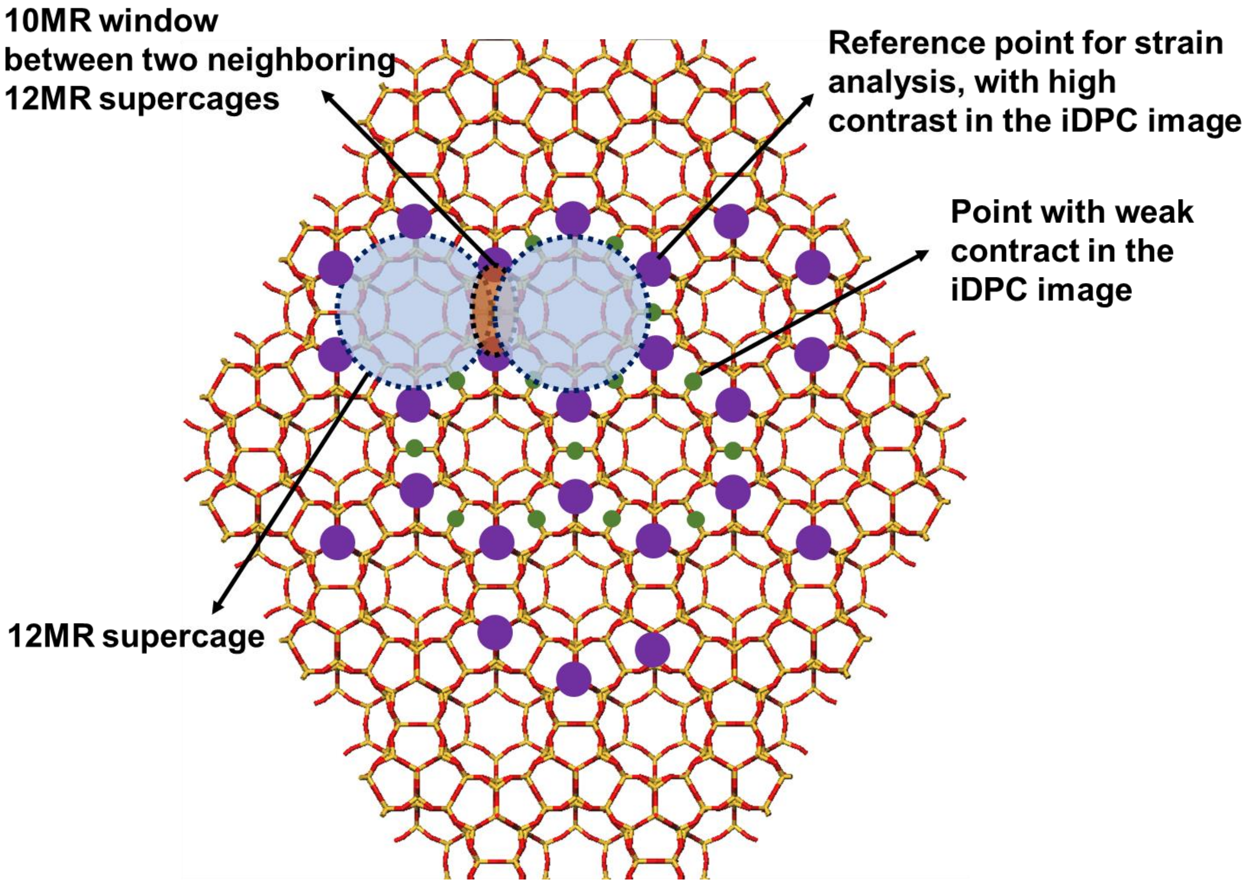

Supplementary Figure 8. Schematic illustration of the position of the reference points (purple) with high contrast in the experimental and the points with weak contrast (green) appeared in the experimental iDPC images. Only the purple points are considered for constructing the network for strain analysis, while the green points are not extracted from the iDPC images.

Supplementary Note 2

The contrast of the Si atomic columns forming the 12MR supercages is much weaker and their detection, for any given single/noise ratio, could only be accomplished with a poorer accuracy, particularly on the experimental images. Therefore, it is not recommendable to use these positions as reference points for strain analysis.

The 6 points used as reference network do not correspond exactly to Si atomic columns in the 12MR supercage, but as shown in the **Supplementary Figure 8**, to positions just outside the supercage (as marked by the purple points). As mentioned previously, these are the points showing the highest contrast in the iDPC images. Nevertheless, the preferential location of Ir species is close to the 10MR windows according to the correlation of the HAADF-STEM and iDPC-STEM images.

Regarding the requirement of adding the centre points to the hexagons in the reference network, it should be emphasized that without this operation, triangulation, using the Delanuy algorithm or any other algorithm, leads to different types of triangles (see **Supplementary Figure 9a**). This complicates the analysis of area changes involved in the calculation of strain, because the initial areas of the triangles will be different, precluding a fair comparison of the variation of the areas. In other words, addition of the hexagon centre allows obtaining the set of identical triangles required for a simple and fair determination of strain, as illustrated in **Supplementary Figure S9b**. In fact, such type of triangulation is the one used in the analysis of strain on more conventional materials.[2,3]

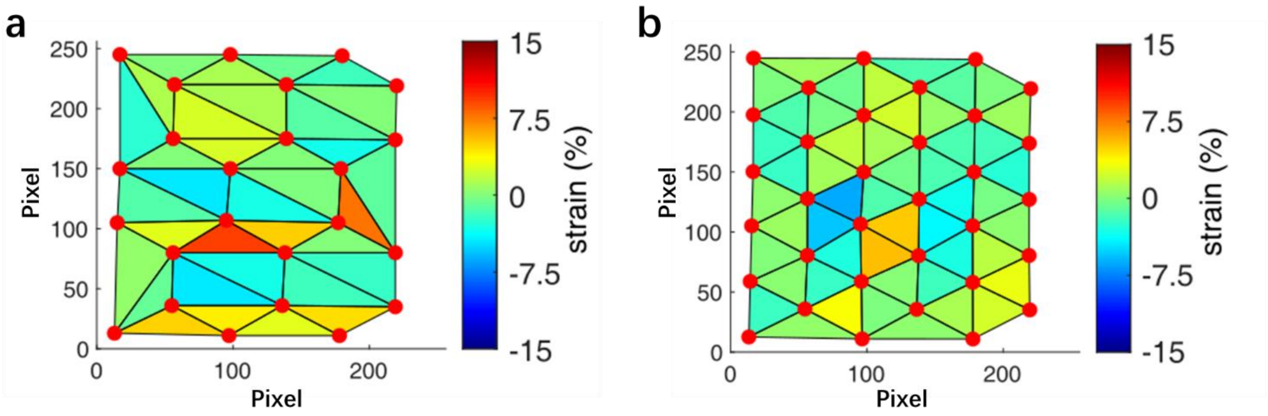

Supplementary Figure 9. Triangulation using Delanuy algorithm without center reference (a) and with center reference (b). The areas of the triangles in (a) are not all identical, which precludes the analysis of the local strain by calculating the variation of the areas. However, after the adding centre points, uniform triangles are formed in the network. The pixel size in this figure is 0.0174 nm.

Supplementary Note 3

iDPC-HAADF STEM image simulation was carried out by using the DFT optimization just in a unit cell of the MWW zeolite. However, to simulate the field of view usually covered in the experimental images, twelve-unit cells have to be considered. Note at this point that a periodic distribution of Ir species is initially obtained when the supercell required for the STEM-iDPC image calculations is created. Such ordered Ir distribution is far away from that observed in the iDPC-HAADF pair images. Therefore, to build models closer to the experimental situation and avoid any problem related to the proximity between neighbouring clusters in the periodic models, only the Si and O positions influenced by only one of the Ir species were modified in the optimized supercell derived from DFT calculations. These supercells, which consider the experimental field of view, are appropriate to check the possibility of detecting the modifications in the positions of Si atomic columns due to Ir species confined in different locations inside de zeolite.

It should also be highlighted at this point that the contrasts due to the Ir species vanish in the iDPC simulated images, regardless their size. This observation is in good agreement with the experimental HAADF-iDPC image pairs, because the contrast in iDPC image is approximately proportional to the atomic number[4]. The large difference in atomic number between Si (Z=14) and Ir (Z=77) and the Z^2-dependence of contrasts in HAADF images allows detecting the location of both isolated Ir atoms and clusters even in reasonably thick zeolite crystallites.

According to the simulated HAADF images, it is possible to distinguish the location of an isolated Ir atom. For instance, a close inspection of the simulated HAADF images of Ir_1-A and Ir_1-D structures indicates that these two different scenarios could be discriminated from the analysis of the position of the contrasts of the isolated Ir atom with respect to the line connecting the contrasts of two adjacent reference network points. In both cases, the contrasts of the Ir_1 single atom locates outside this line, but in the Ir_1-A case, the projection of these contrasts on this line take place on a point closer to one of the ends.

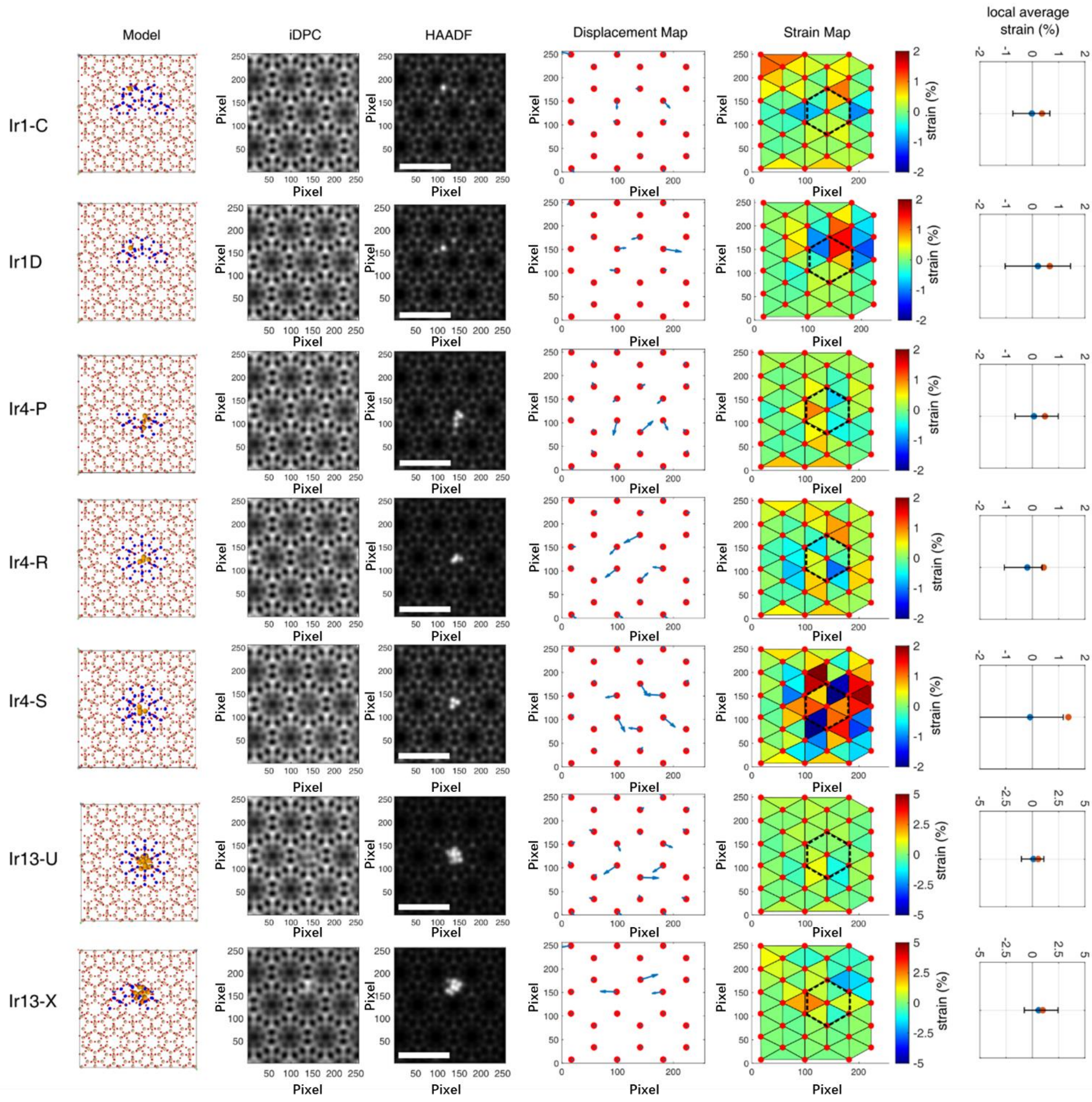

Supplementary Figure 10. **Image simulation results of various Ir@MWW structures.** The processing of the models is described in the workflow illustrated in **Figure 2**. In these models, the blue atoms are marked as the Si and O positions influenced by one iridium atom or cluster. Based on these models, the iDPC and HAADF images are simulated and presented. The displacement of the reference point in the iDPC image are obtained after comparing the simulated image with the theoretical structure of pure-silica MWW zeolite. Delanuy triangulation on the structural feature generated according to the simulated iDPC images and strain distribution for various models are obtained based on the calculations described in **Figure 2**. In the right column, the strain range spanned by these triangles (segment bars) as well as the average of local strain values (blue dots) and the average of the absolute

strain values (orange dot in the plots) are shown. The pixel size in this figure is 0.0174 nm and the scale bar is 2 nm.

Supplementary Note 4

Geometric phase analysis (GPA) has become a general method to quantify the local strain in nanostructured materials by using in general HREM images. This method is based on measuring the lattice distortions by transforming the information recorded in the image to the spatial frequency space. This transformation is made by performing Fast Fourier Transforms (FFTs) on areas of the same image where some distortion is expected and these FFTs are compared to those obtained in distortion-free areas. However, it has been reported that this method introduces artefacts and the spatial resolution is limited to a unit cell.[5,6]

An additional difficulty which arises in the particular case of zeolite materials is the impossibility to select a distortion-free area as the reference for GPA because of the defective sites generated during the hydrothermal synthesis of the Ir-zeolite materials, as we have mentioned in the original manuscript. Moreover, the presence of subnanometric Ir species distributed throughout the zeolite crystallites makes it unfeasible to choose fully relaxed areas which can be used as a reference.

Alternatively, other methods have been developed to quantify local strain in nanomaterials using aberration-corrected STEM images. These methods are mainly based on processing the information obtained in the real space to estimate with high accuracy the location of the atomic columns. This estimation is made by calculating the centre of mass of the atomic-column like image contrasts and then refining the initial coordinates by fitting them to a 2D-Gaussian pattern.[7,8] Though it has been reported that this methodology can be influenced by the scanning distortion, Nord *et al.* have demonstrated that the effect of the instabilities can be fruitfully avoided by acquiring fast images, *i.e.* by using a very short dwell time (in the order of 2 μs). In the case of conventional HAADF-STEM images, the signal-to-noise ratio of images recorded under these conditions is very low and the way to improve it involves acquiring a series of consecutive fast images of the same area. Then, these images must be post-processed to improve the imaging quality and the sample should remain stable under the e-beam irradiation during the collection of multiple HAADF-STEM images.

In this work, we have simultaneously recorded, in pairs, HAADF and iDPC-STEM images with a dwell time of 1.25 μs, providing an overall recording time of only 7 s for the paired HAADF and iDPC images. Using this very fast scanning mode, we can avoid the problem of sample drift or instabilities. The advantage of recording iDPC-STEM images is that the signal-to-noise-ratio is high, even for those recorded under the fast-scanning conditions.[9] Moreover, it should be noted that, the strain analysis on the zeolite framework structure along this work has been performed with the iDPC images, which provide much higher spatial resolution than the conventional HAADF images. The role of HAADF

images are used to determine the local of Ir species because of the higher contrast of Ir than the zeolite framework in the HAADF imaging mode.

To evaluate the accuracy of the proposed methodology, we have determined the x−y coordinates of all the intensity maxima observed in the simulated iDPC image of a non-distorted zeolite and compared them with those derived from the positions of the atomic columns of the zeolite structure refined by DFT. After applying the above procedure, the positions determined for the atomic columns in the simulated iDPC image are in fact quite close to those in the models, within distance differences of 1.3 pm on average. The analysis of local strain in the simulations of distorted models leads to displacements of the image contrasts in the order of 14 pm, which is one order of magnitude higher than that detected for the non-distorted model. Moreover, the analysis of distortions in the experimental images leads to values in the same range of the simulated distorted models, giving us the confidence to use the experimental iDPC images to measure and calculate the local strain in the zeolite framework.

Based on the above analysis, we think the methodology developed in this work can give reliable evaluation on the local strain of the MWW zeolite framework.

Supplementary Note 5

Zeolites are vulnerable under e-beam and the e-beam induced damage is related to the acceleration voltage and electron dose on these materials.[5] To reduce the damage, all the images presented in this study were recorded at 300 kV to minimize the radiolysis-induced damage to the zeolite framework.

To avoid beam damage effects and beam-induced modifications of the pristine zeolite structure, we have worked under low-dose imaging conditions along the whole study. In particular, 2048 × 2048 images, with a pixel size of 17.43 pm, beam current of 10 pA and dwell time of 1.25 μs were recorded for the image analysis and calculations. Using these experimental conditions, the electron dose amounts to 2570 e-/$Å^2$. Previous works indicate a dose in the order of 5000 e-/$Å^2$ as the threshold to induce beam damage in pure-silica zeolite materials (e.g. MFI-type zeolite). Though this threshold value must be very likely dependent on the zeolite topology, our dose was roughly 50% lower and the pure-silica MWW zeolite structure should has similar stability as the MFI-type zeolite under the imaging conditions. Therefore, we are confident that in a single HAADF-iDPC paired image, the atomic positions of the zeolite atomic columns should not be modified.[10,11]

To check the stability of the MWW zeolite structure, additional experiments were performed. In particular, experiments in which three consecutive HAADF-iDPC pair images were recorded. A series of areas were explored using this approach (as show in **Supplementary Figure 11-15**). The analysis of the whole set of the three consecutive iDPC-STEM image pairs revealed no significant change after the first shot. Neither the location of the Ir clusters nor the corresponding iDPC image is altered in the first two consecutive iDPC-STEM images. In some areas, the MWW zeolite framework is partially damaged in the third scan, as shown in **Supplementary Figure 13f** and **Supplementary Figure 15f**.

Nevertheless, it should be mentioned that the strain analysis of the experimental HAADF-iDPC image pairs is performed by cropping local areas of interest (areas with high imaging quality) from the entire image (image size 2048 × 2048 pixel). By using this approach, we avoid also exposing the zeolite to a high total dose of electrons. To illustrate this, we show below a typical iDPC image with a full size in the 30-40 nm range recorded in our studies (see **Supplementary Figure 16**). The coloured squares (~4 nm) mark the areas where the strain analysis was performed. In other words, we have used intermediate magnifications during image recording, in order to avoid depositing a large number of electrons in small areas.

Following the Reviewer's comment, we have included the additional images in the revised Supplementary Information to show the stability of the MWW zeolite structure during the recording of first two consecutive images.

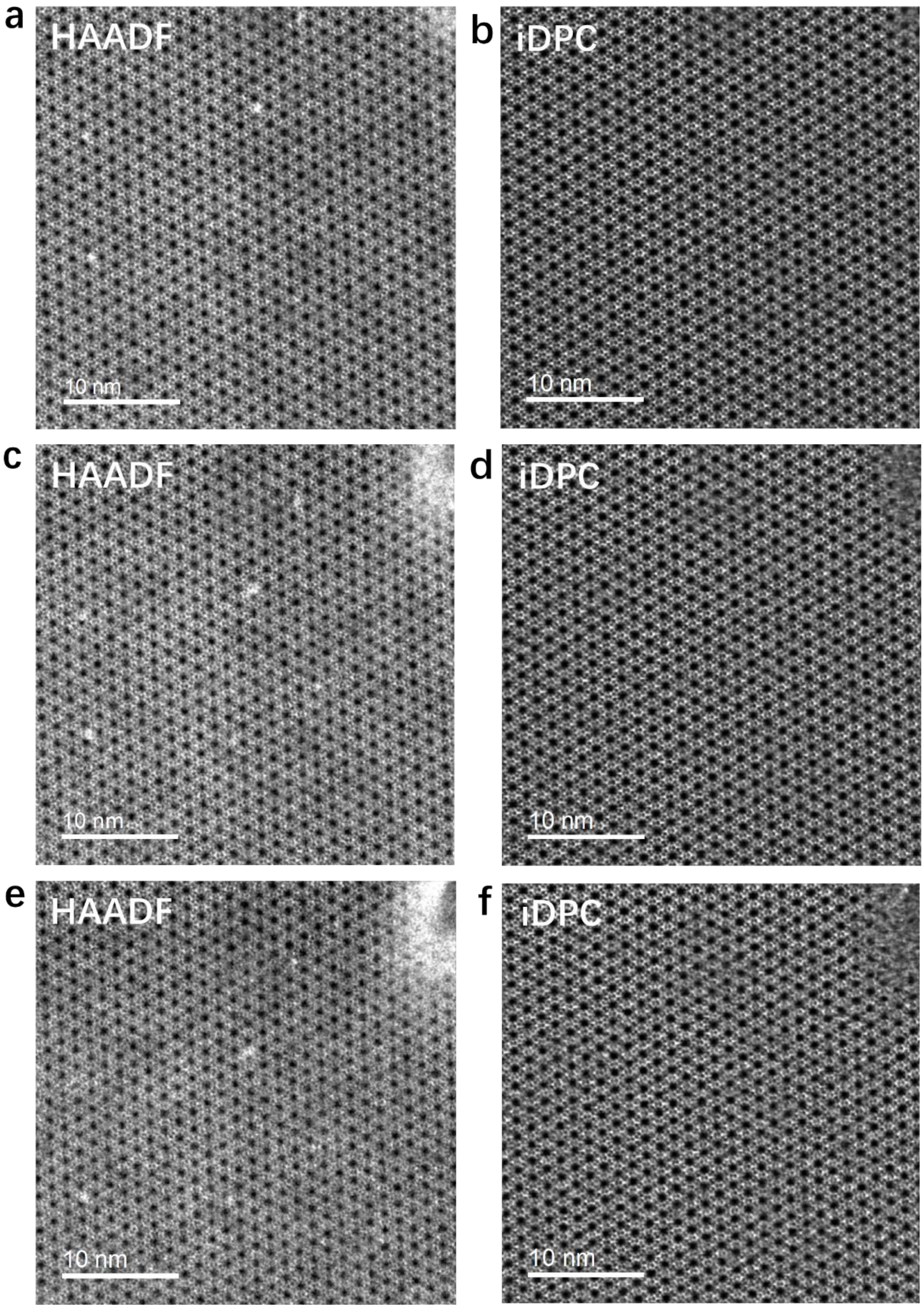

Supplementary Figure 11. Consecutive recording of paired HAADF-STEM and iDPC-STEM images of the Ir@MWW-subnano sample to test the stability of the zeolite structure under the e-beam. (a, b) First scan, (c, d) second scan and (e, f) third scan. The imaging field is manually shifted in a small distance (<5 nm) because the centre the e-beam will cause the damage of the zeolite, as reflected in the upright corner. (a, c, e) HAADF-STEM images and (b, d, f) the corresponding iDPC images.

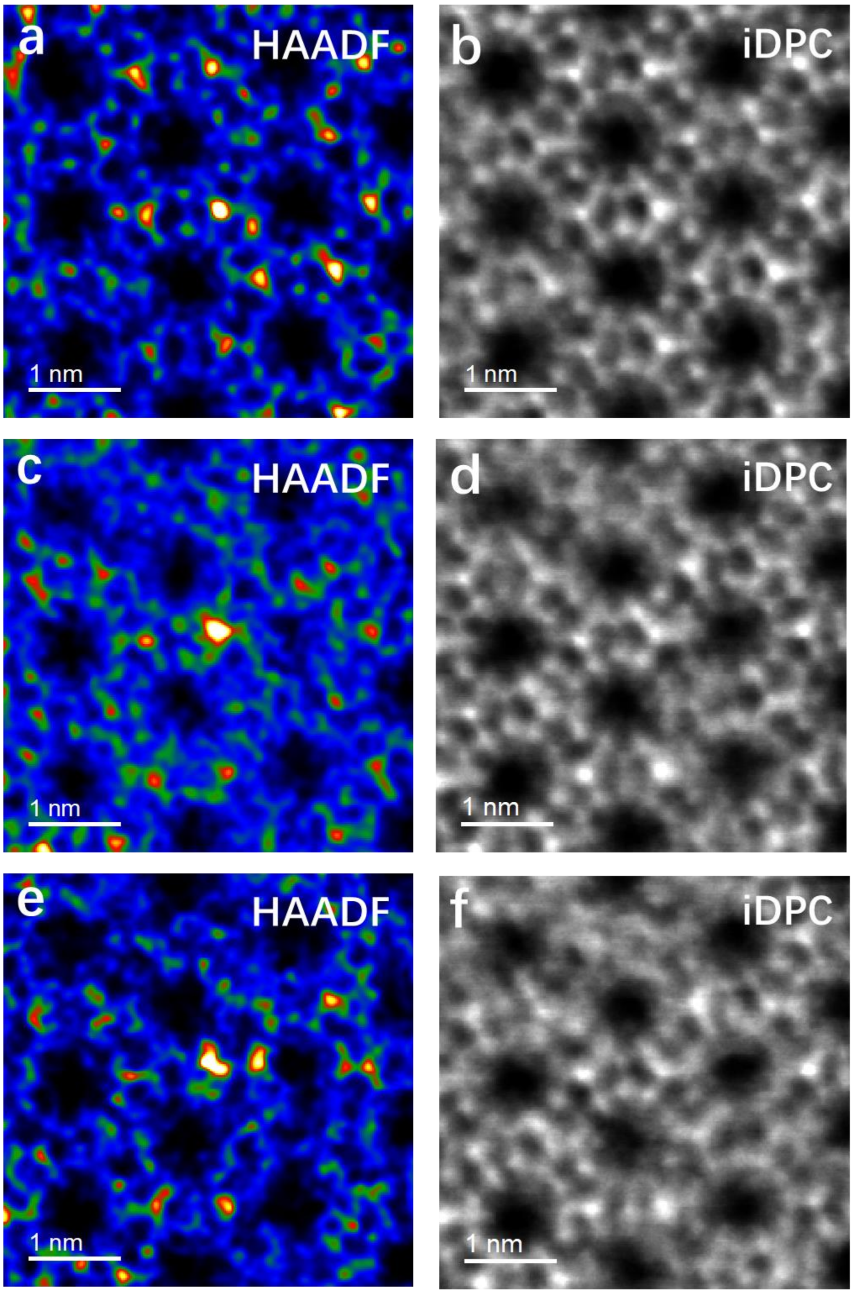

Supplementary Figure 12. Consecutive recording of paired HAADF-STEM and iDPC-STEM images of the Ir@MWW-subnano sample to test the stability of the zeolite structure under the e-beam. The local images were cropped from the images shown in **Supplementary Figure 11** to show the detailed structures of the Ir species and the surrounding zeolite framework. (a, b) First scan, (c, d) second scan and (e, f) third scan. The imaging field is manually shifted in a small distance (<5 nm) because the centre the e-beam will cause the damage of the zeolite, as reflected in the upright corner. (a, c, e) HAADF-STEM images and (b, d, f) the corresponding iDPC images.

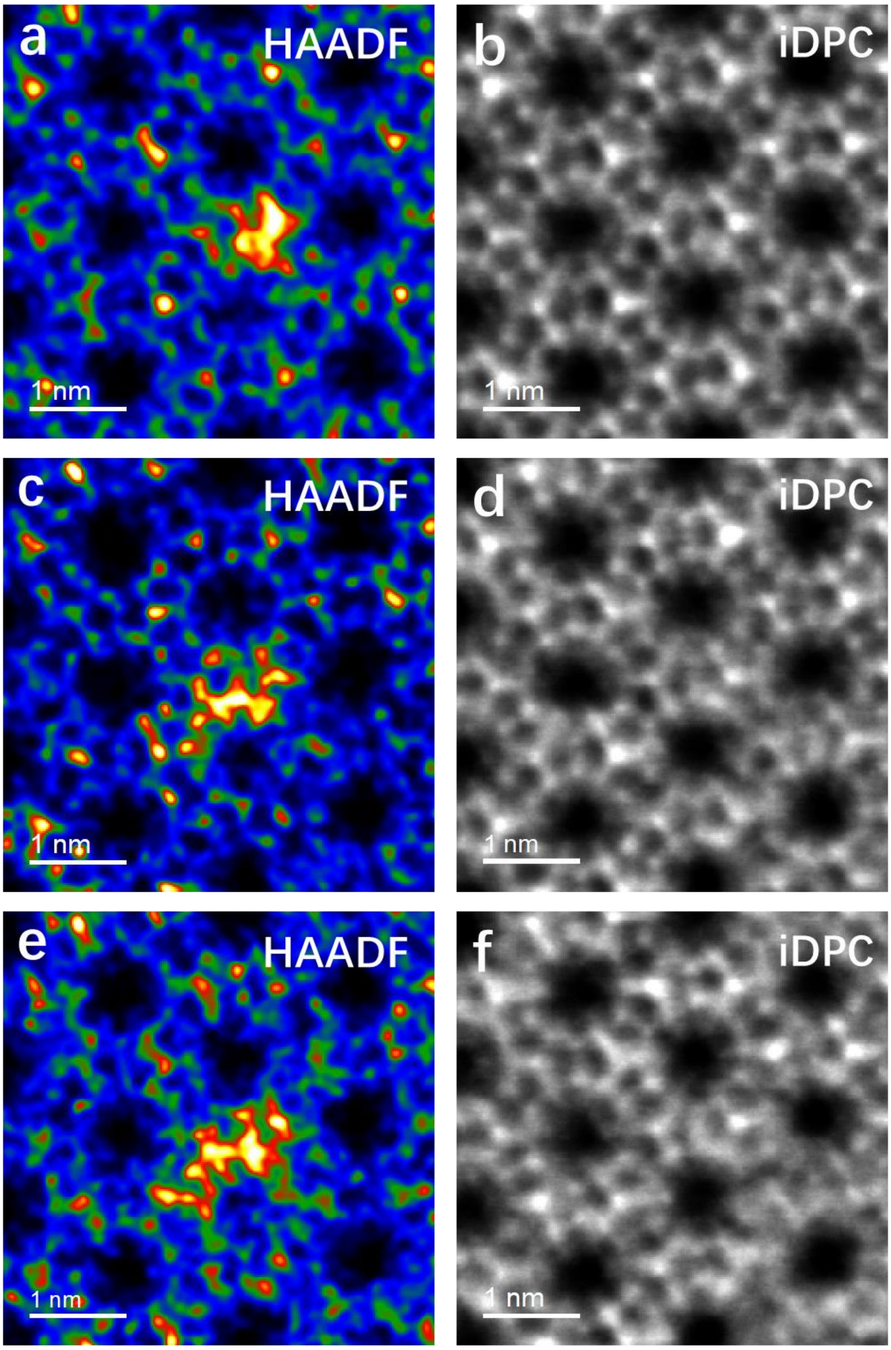

Supplementary Figure 13. Consecutive recording of paired HAADF-STEM and iDPC-STEM images of the Ir@MWW-subnano sample to test the stability of the zeolite structure under the e-beam. The local images were cropped from the images shown in **Supplementary Figure 11** to show the detailed structures of the Ir species and the surrounding zeolite framework. (a, b) First scan, (c, d) second scan and (e, f) third scan. The imaging field is manually shifted in a small distance (<5 nm) because the centre the e-beam will cause the damage of the zeolite, as reflected in the upright corner. (a, c, e) HAADF-STEM images and (b, d, f) the corresponding iDPC images.

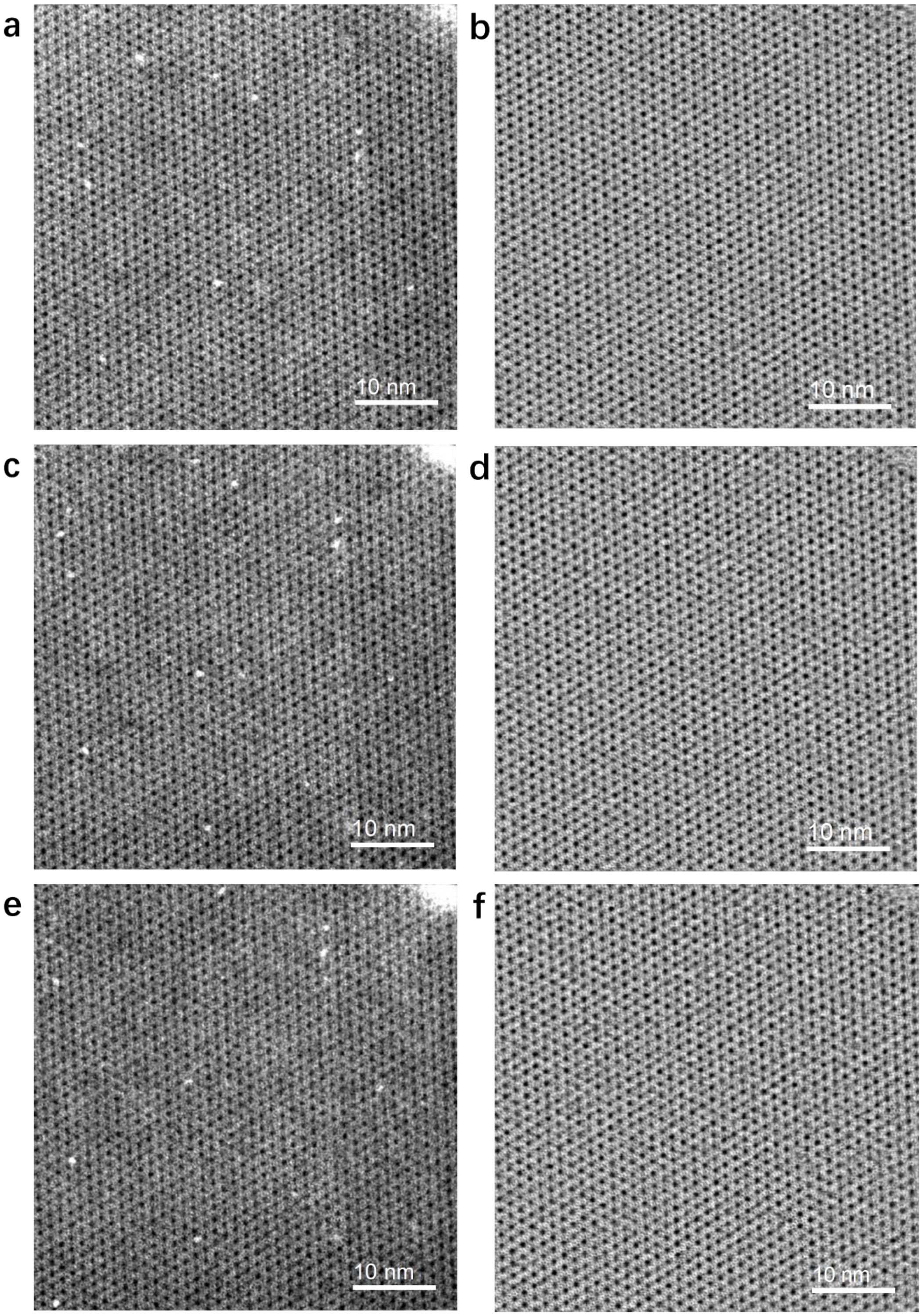

Supplementary Figure 14. Consecutive recording of paired HAADF-STEM and iDPC-STEM images of the Ir@MWW-subnano sample to test the stability of the zeolite structure under the e-beam. (a, b) First scan, (c, d) second scan and (e, f) third scan. The imaging field is manually shifted in a small distance (<5 nm) because the centre the e-beam will cause the damage of the zeolite, as reflected in the upright corner. (a, c, e) HAADF-STEM images and (b, d, f) the corresponding iDPC images.

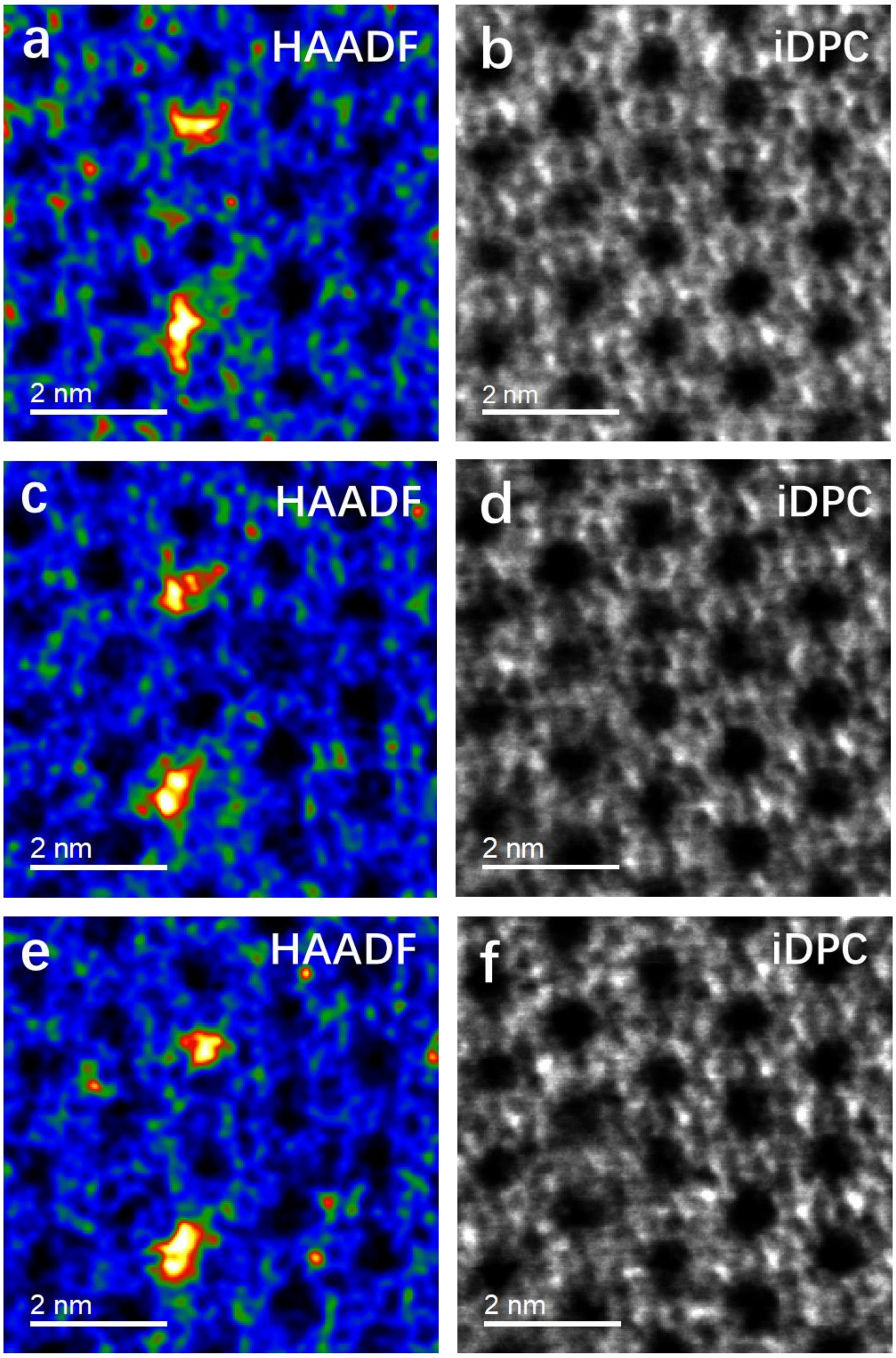

Supplementary Figure 15. Consecutive recording of paired HAADF-STEM and iDPC-STEM images of the Ir@MWW-subnano sample to test the stability of the zeolite structure under the e-beam. The local images were cropped from the images shown in **Supplementary Figure 14** to show the detailed structures of the Ir species and the surrounding zeolite framework. (a, b) First scan, (c, d) second scan and (e, f) third scan. The imaging field is manually shifted in a small distance (<5 nm) because the centre the e-beam will cause the damage of the zeolite, as reflected in the upright corner. (a, c, e) HAADF-STEM images and (b, d, f) the corresponding iDPC images.

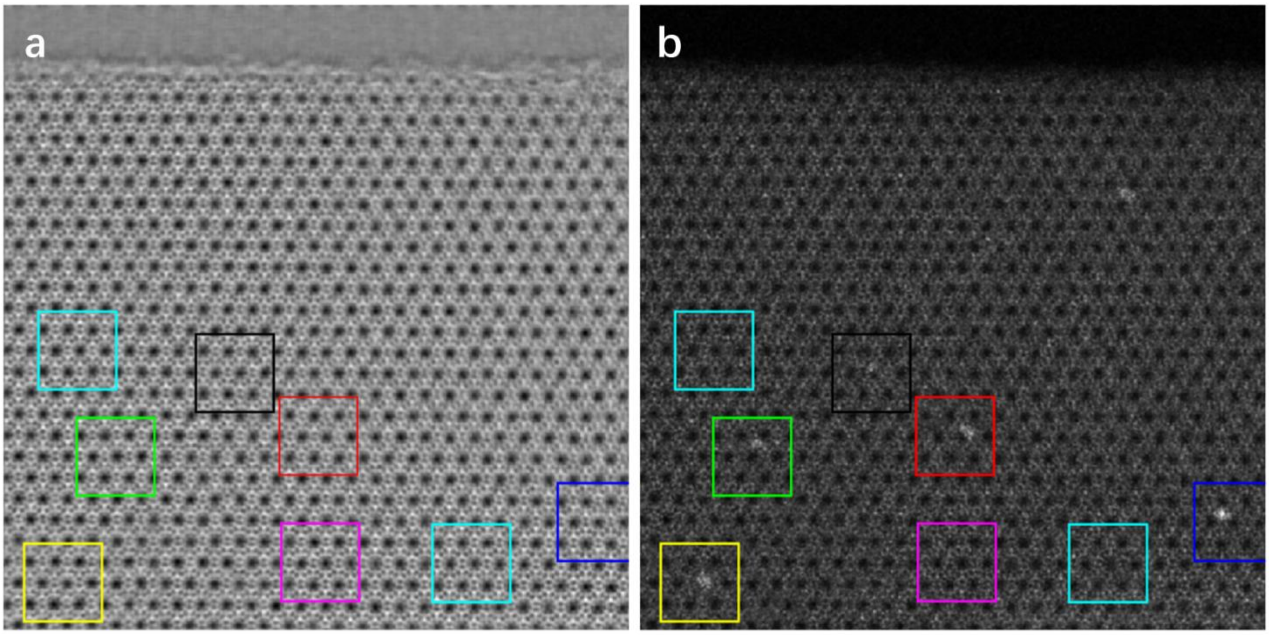

Supplementary Figure 16. Typical paired iDPC-STEM (a) and HAADF-STEM (b) images used for the strain analysis. A large image is recorded in which several areas (as marked by the coloured squares) with high imaging quality are chosen for further strain analysis.

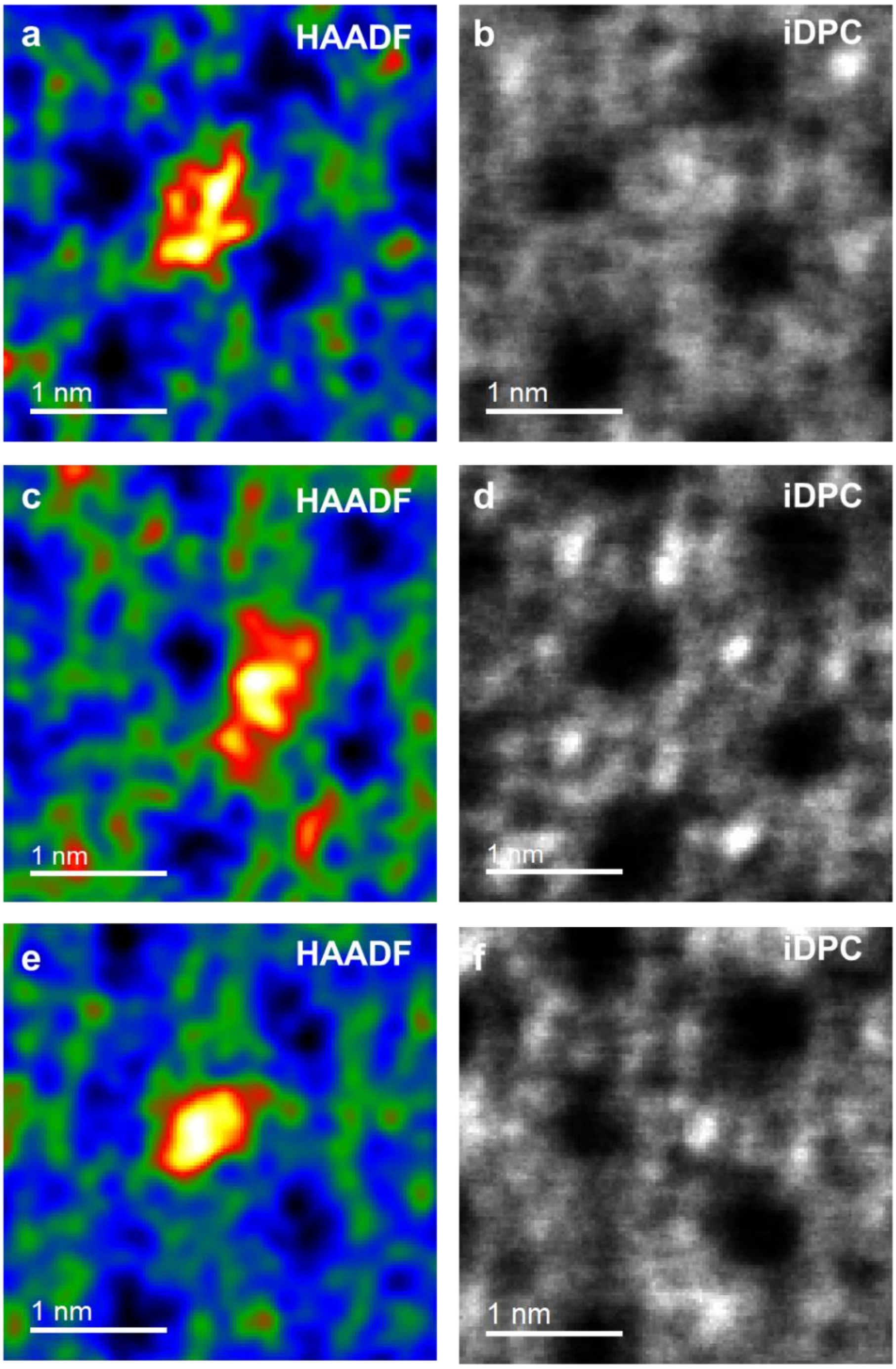

Supplementary Figure 17. Structural characterization of Ir@MWW-subnano sample. In this sample, the vast majority of the Ir species are Ir clusters, located at the 10MR window connecting the 12MR supercages, as determined by the HAADF-STEM (a, c and e) and the corresponding iDPC-STEM (b, d and f) paired images.

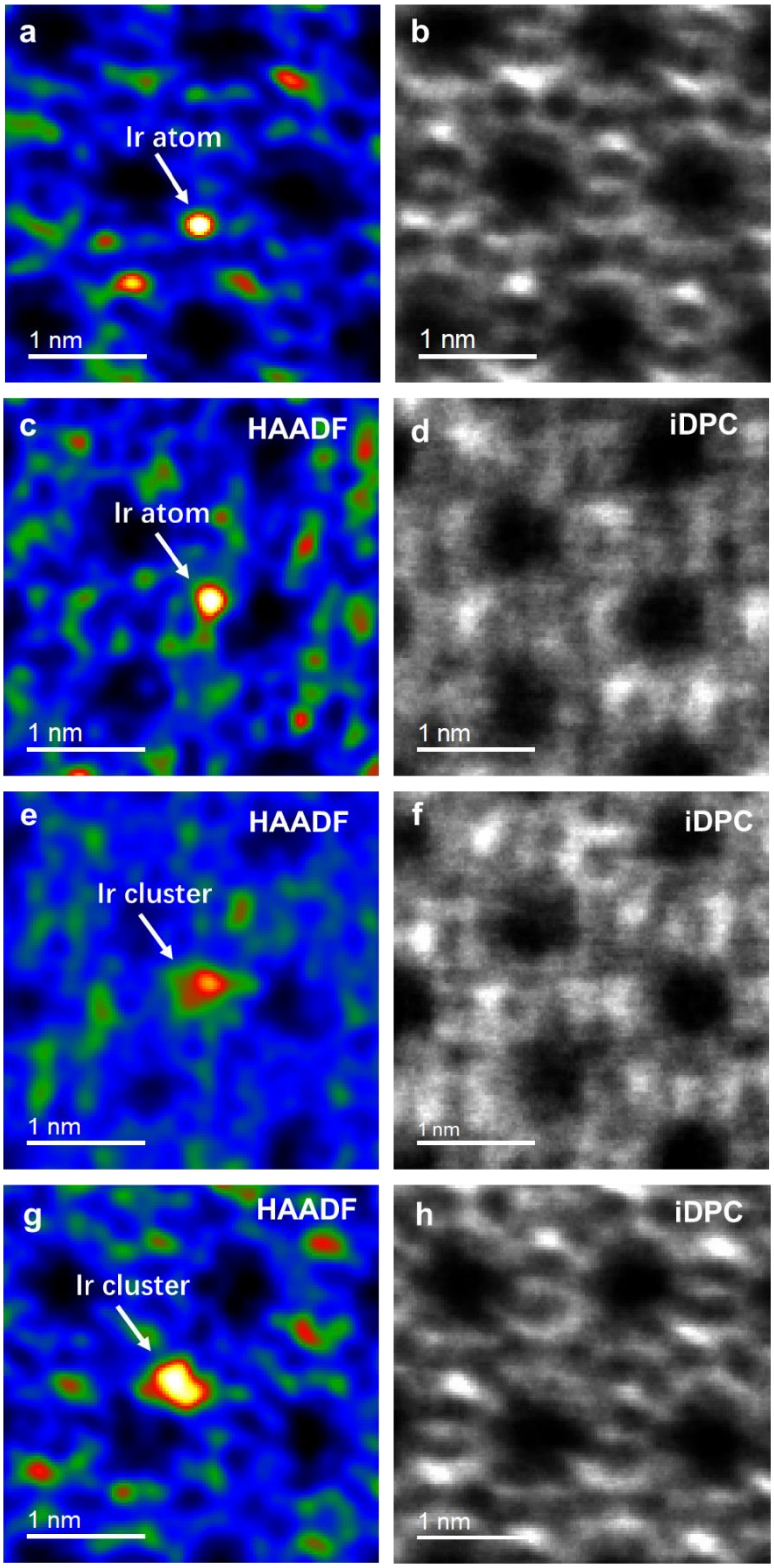

Supplementary Figure 18. Structural characterization of Ir@MWW-subnano sample. In this sample, a small fraction of the Ir species exist as isolated Ir atoms (a-d) and very small Ir clusters (e-h), as shown in this figure. By measuring multiple areas of the Ir@MWW-subnano sample, we are able to study the size-dependent local strain the MWW zeolite structure caused by the Ir-zeolite interaction.

Supplementary Note 6

The TEM and STEM images are two-dimensional projection images. In particular, the depth of field is roughly 8 nm under our imaging conditions and this depth implies that clusters/atoms within these distances may be imaged in a single shot. Since the distances between sites along the incoming beam direction may be in the order of 3 nm (depending on the exact location), two metal species could be imaged as a single one. Though this cannot be neglected, the very low Ir loading in the Ir@MWW-subnano sample (0.24 wt% of Ir in pure-silica MWW, corresponding to a Si/Ir atomic ratio of >1300) makes it quite unlikely to occur. Thus, a simple calculation taking into account of the Ir loading and the density of the MWW zeolite indicates that we can expect less than 5 Ir atoms in the volume corresponding to 3 × 3 × 3 unit cells of the zeolite (corresponding to a volume of 4.2 × 4.2 × 7.2 nm^3).

Concerning the estimation of the thickness of sample, we agree with the Reviewer that in many materials, it can be simply achieved by EELS or CBED. However, zeolites cannot withstand the dose conditions required for these experiments, especially under the circumstance that these experiments have to be followed by recording the necessary HAADF-iDPC image pairs.

To have an idea on the morphology and thickness of the MWW zeolite crystallites, we have measured the Ir@MWW-subnano sample by FESEM. As shown in the FESEM image, the MWW zeolite crystallites show morphology close to hexagonal plates. A value in the order of 10-30 nm was observed in these cases (see **Supplementary Figure 19**). Considering the low Ir loading in the Ir@MWW sample, it can be concluded that, the overlapping of two Ir species along the [001] direction in the MWW zeolite crystallite will be very rare.

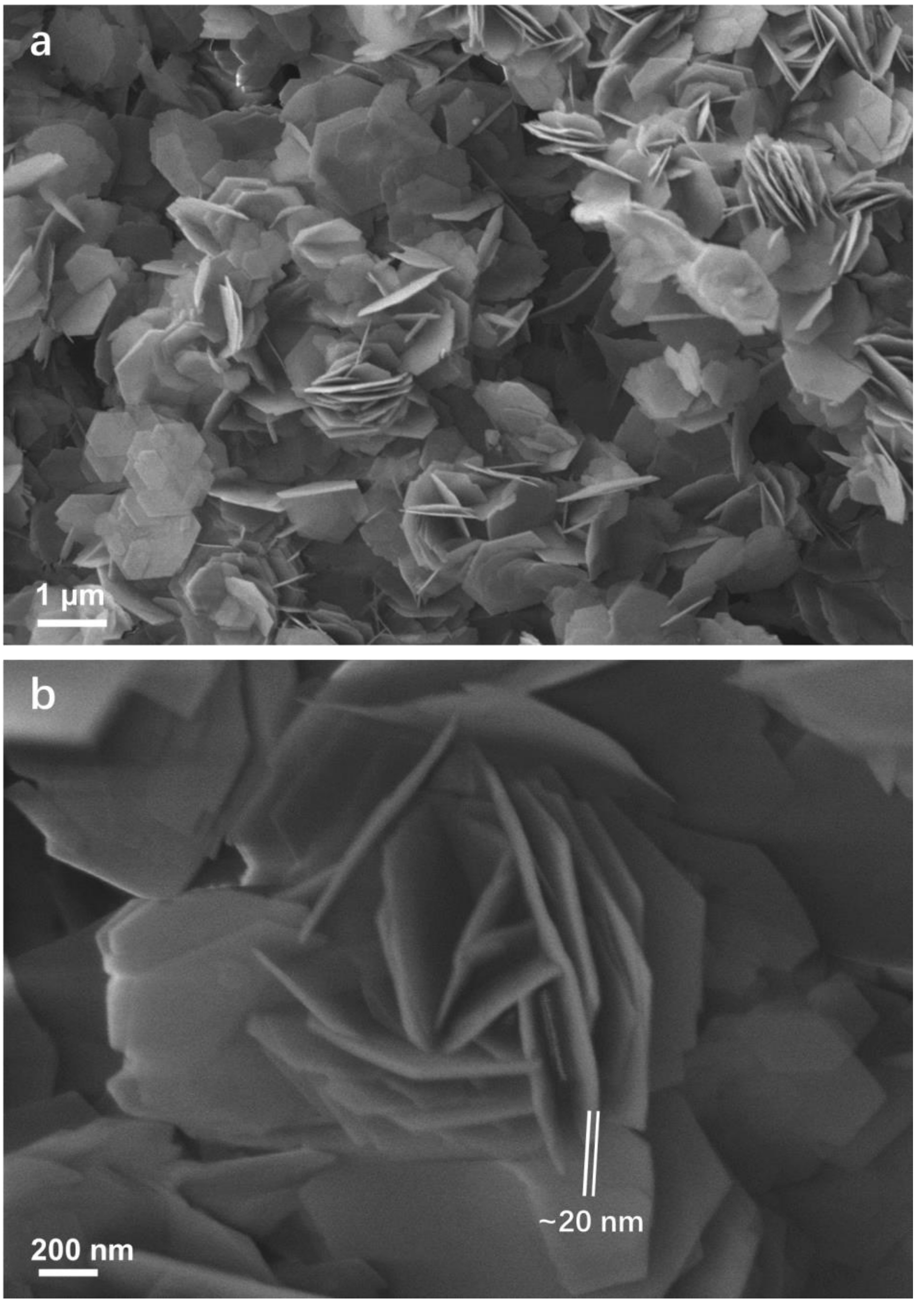

Supplementary Figure 19. Field-emission scanning electron microscopy images of Ir@MWW-subnano sample. The MWW zeolite crystallites show two-dimensional morphology and the thickness of the zeolite crystallites are mostly between 10-30 nm.

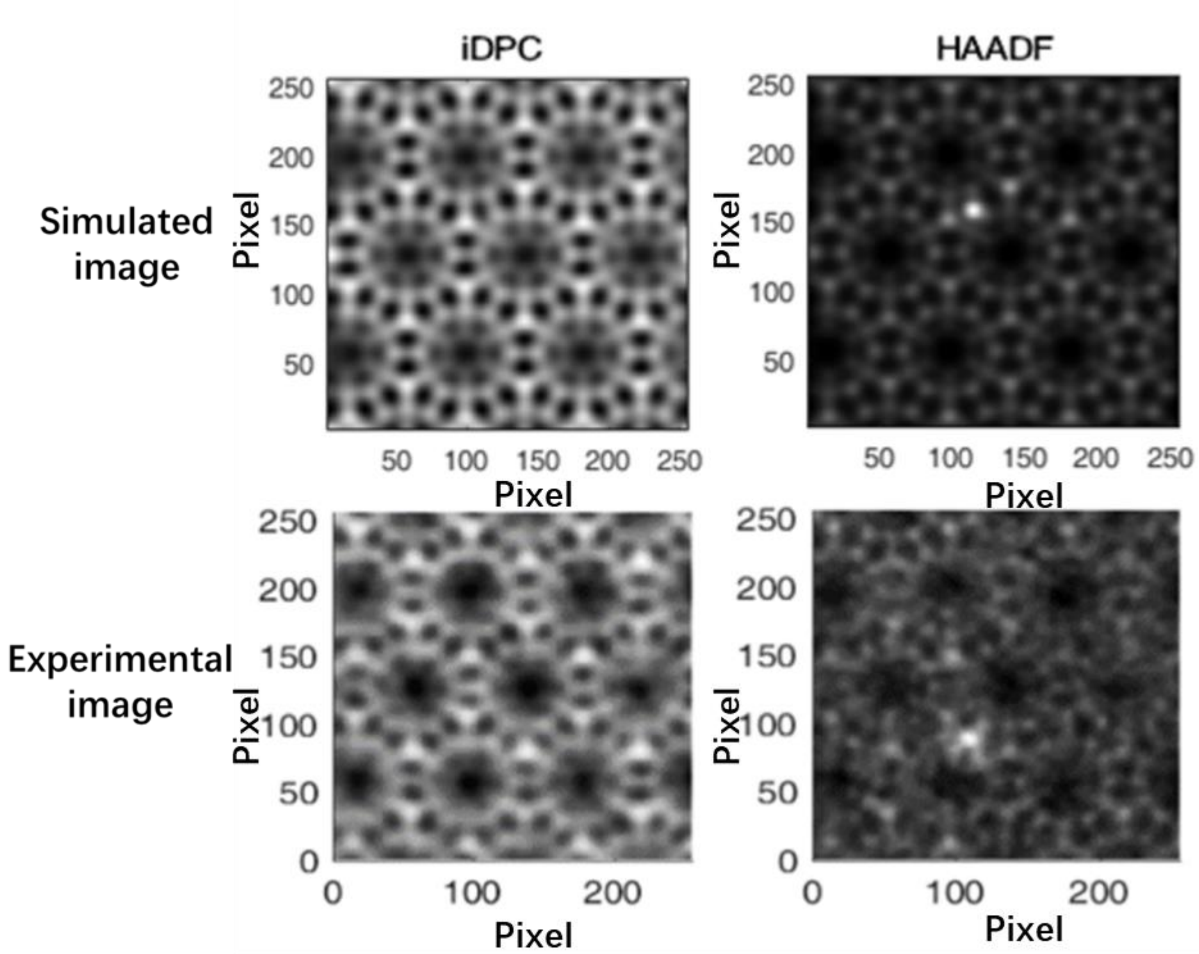

Supplementary Figure 20. (Top) Representative iDPC-HAADF image pair simulation for structure Ir_1-C. (Bottom) experimental iDPC-HAADF image pair recorded with the Ir@MWW-subnano sample. In the Ir_1-C structure, the Ir atom is located at the 5MR unit of the 10MR window. The experimental HAADF and iDPC images are well matched with the simulated images. The pixel size in this figure is 0.0174 nm.

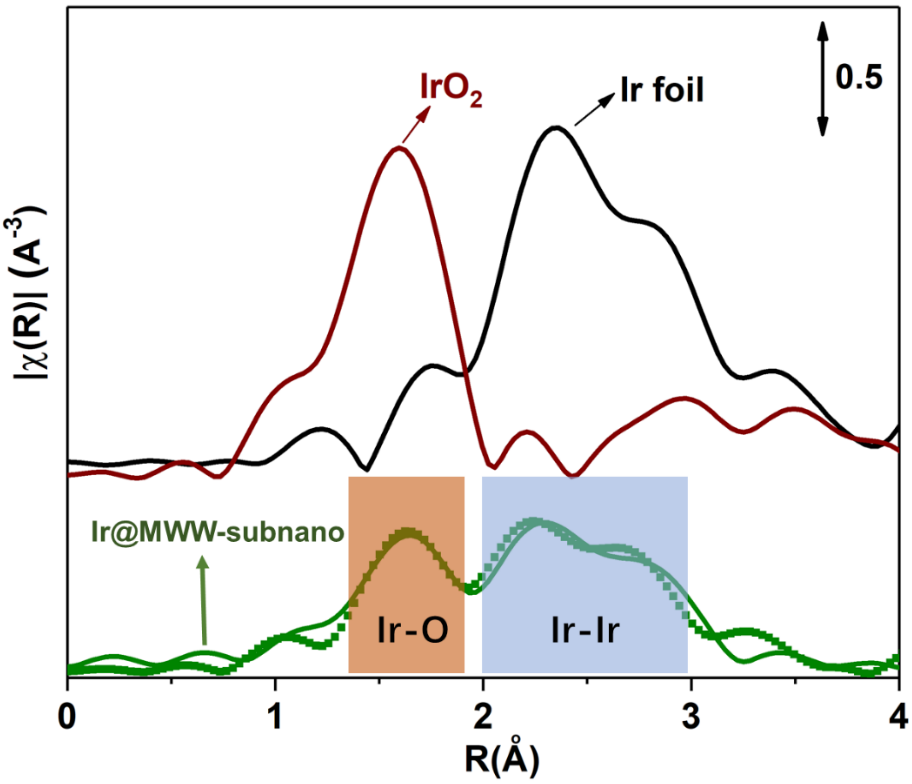

Supplementary Figure 21. EXAFS spectra of the Ir@MWW-subnano sample and the IrO_2 and Ir references. As shown in this figure, both Ir-O and Ir-Ir contribution can be observed in the EXAFS spectrum of the Ir@MWW-subnano sample[12]. The Ir-O contribution can be ascribed to the Ir-O bonding between the isolated Ir atoms and the zeolite framework and also to the Ir-O bonding between the Ir cluster and zeolite framework. The Ir-Ir contribution is associated to the metallic bonding within Ir clusters.

It should be noted that, the majority of Ir species in the Ir@MWW-subnano sample is low, because most of the Ir atoms are in the form of Ir clusters, as observed in the HAADF-STEM images. The Ir-edge EXAFS spectra will reflect the averaged information of all the Ir species in the whole material. Therefore, due to the low percentage of isolated Ir atoms in the Ir@MWW-subnano sample, the contribution of the Ir-Si bonding in the Ir-edge EXAFS spectra is too low to be observed.

Supplementary Note 7

As can be seen in **Figure 4** shown in the main text, a fairly good linear correlation between the local absolute strain measured in the experimental images and the charge of the Ir species derived from theoretical calculations is presented. Notably, in the data points whose net atomic charge is larger than -0.2 e, the linear correlation is clearly better. Some data points whose net atomic charge is close to 0 show deviations from the fitting plot. In those cases, local strain values are also not high, implying that the interaction between the metal species and the zeolite framework is not strong in those structure configurations.

According to the principles of the measurement of local strain, the local strain is caused by the distortion of the zeolite framework induced by the metal-zeolite interaction. Regarding the net atomic charge, it can be understood as a result of the overall electronic interaction between the metal species and zeolite framework. In the case of Ir_4 and Ir_{13} clusters, several chemical bonding interactions are formed according to the theoretical calculations, which lead to local distortion in the zeolite framework. However, in some configurations, the sum of the charge transfer of several chemical bonding could result a low net atomic charge in the Ir cluster, because the electronic transfer in one bonding interaction could be compensated by another bonding interaction with reverse charge transfer. Consequently, slight deviations are observed in the linear correlation between the local strain and net atomic charge, as shown in **Figure 4**, especially for the structure configurations giving low local strain values.

Despite the slight deviations in a few cases, the general trend clearly indicates an interconnection between the geometric and electronic structures of metal-zeolite materials.

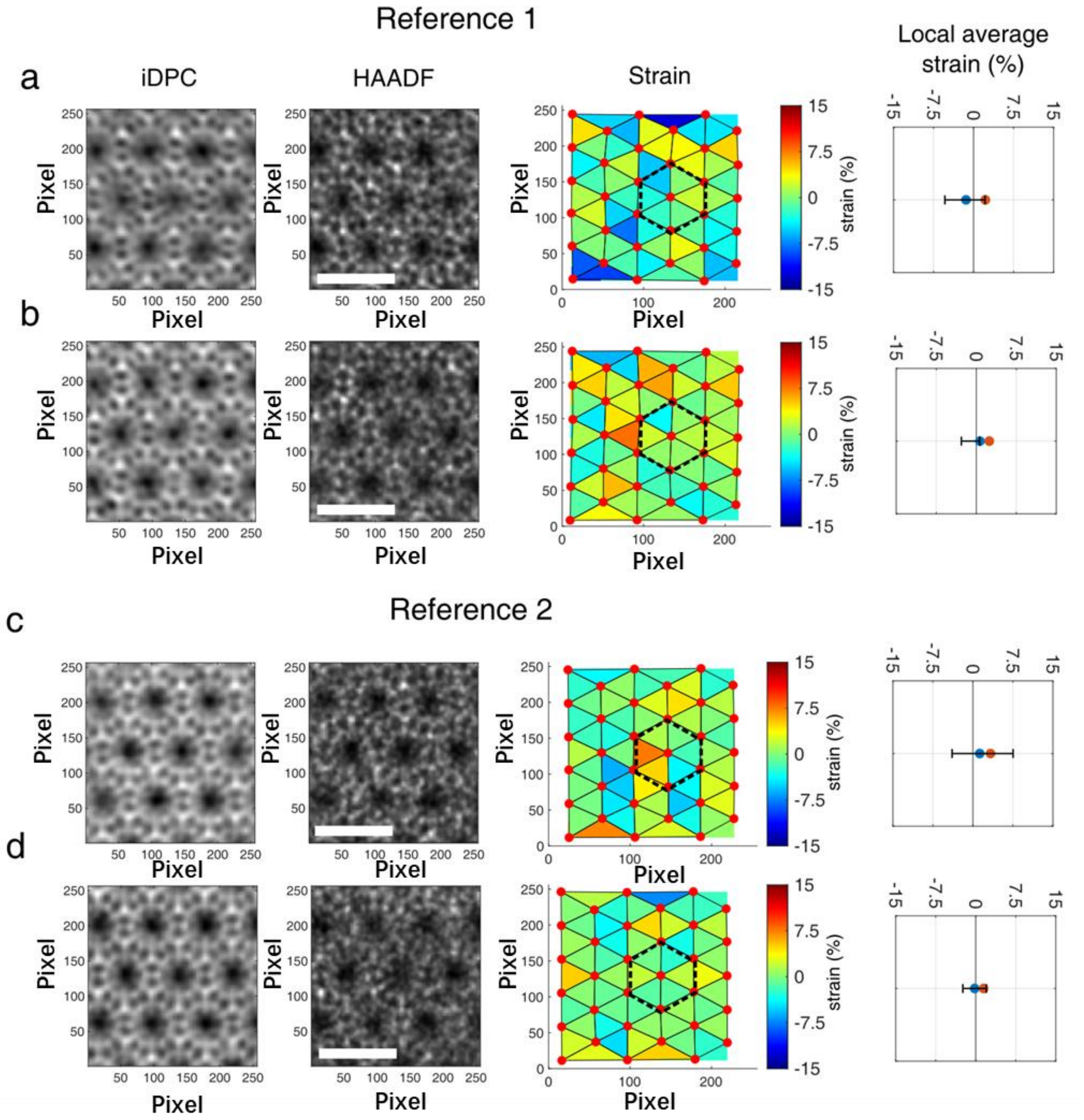

Supplementary Figure 22. Strain analysis of references areas in the experimental iDPC-HAADF STEM image pair. In the right column, the strain range spanned by these triangles (segment bars) as well as the average of local strain values (blue dots) and the average of the absolute strain values (orange dot in the plots) are shown. The pixel size in this figure is 0.0174 nm and the scale bar is 2 nm.

According to our previous work, the conditions used to record the STEM images provide a depth of field of ~8 nm, indicating that in the areas used as a reference there is no other Ir species at a distance

lower than the imaging depth[10]. Even in those models with the largest E_{def}, the structural modification induced by the Ir species does not propagate to such large distances, as discussed in the main text and shown in **Figure 5**. Instead, the confinement effects are just reflected on the shift of the atomic columns in the neighbouring areas of Ir species. Therefore, the residual strain measured in Ir-free areas of the image could be caused by the intrinsic defects in the zeolite structure (such as silanol groups) or to the intrinsic experimental errors of the method (for instance, errors caused by the image acquisition).

As shown in **Supplementary Figure S22a**, strain spans in this case from -5.4% to +2.2%. The average strain (blue dot) amounts to -1.4% and the average of the absolute values of strain (orange dot) is +2.2%. This analysis was performed in a second Ir-free area of the same crystal (**Supplementary Figure S22b**). Both magnitudes deviate from the zero-value expected for an ideally perfect MWW crystallite. However, as previously mentioned, the intrinsic errors of the method, the presence of local defects (could be induced by the synthesis, by hydration or hydroxylation of the crystallites or by beam-damage effects) or a possible long-range influence of other Ir species located at distances beyond the depth of field could explain these residual strain values in the measured area.

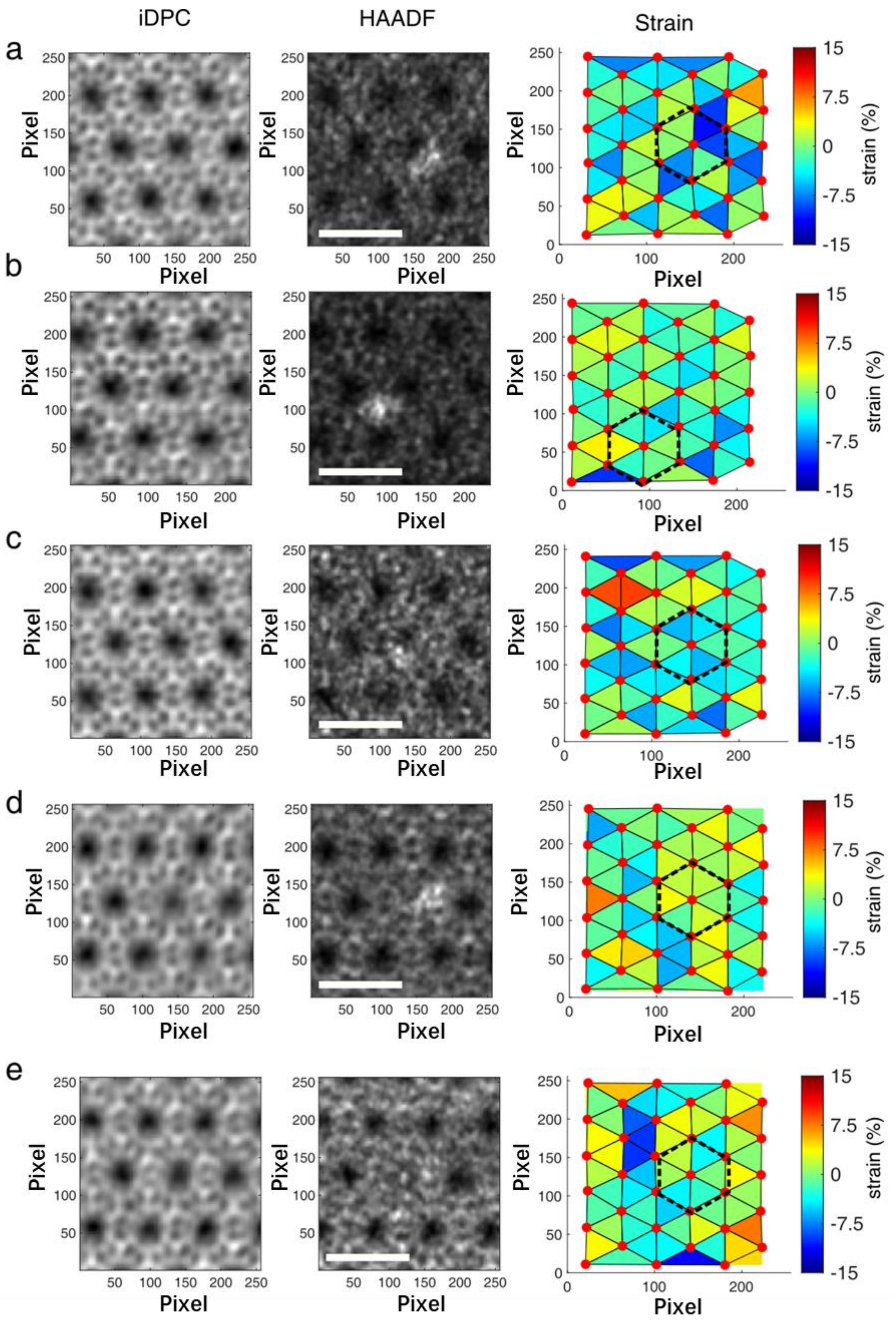

Supplementary Figure 23. Experimental measurements of the local strain in Ir@MWW-subnano sample. In this figure, more paired HAADF and iDPC images have been recorded and

analysed to show the local strain of Ir clusters located in MWW zeolite. Besides the cases with Ir clusters located at the 10MR windows (shown in panels a-c), two areas with Ir clusters located at the 12MR supercage are also measured (shown in panels d and e). Notably, the cases comprising Ir clusters located at the supercage give low local strain values, which is well consistent with the theoretical results. The pixel size in this figure is 0.0174 nm and the scale bar is 2 nm.

Supplementary Table 1. Values of local net strain and absolute strain before and after off-set correction

	Local Net Strain (%)	Off-set corrected Local net strain (%)	Local Absolute Strain (%)	Off-set corrected Local Absolute Strain (%)
**REF**	**-0.6**	0.0	**1.9**	0.0
**Figure 5a**	0.4	1	3.3	1.4
**Figure 5c**	1.2	1.8	2.6	0.7
**Figure 5d**	1.0	1.6	2.5	0.6
**Figure S14d**	1.4	2.0	1.7	-0.2
**Figure 5e**	2.5	3.1	2.5	0.6
**Figure S14e**	-1.2	-0.6	2.1	0.2
**Figure 5g**	-2.7	-2.1	3.8	1.9
**REF**	**-1.4**	0.0	**2.1**	0.0
**Figure S14a**	-4.5	-4.0	4.9	2.8
**Figure S14b**	-2.1	-1.6	2.8	0.8
**Figure 5b**	-2.24	-1.74	5.3	3.3
**Figure S14c**	-3.6	-3.1	3.6	1.6
**Figure 5f**	-1.6	-1.1	2.4	0.4

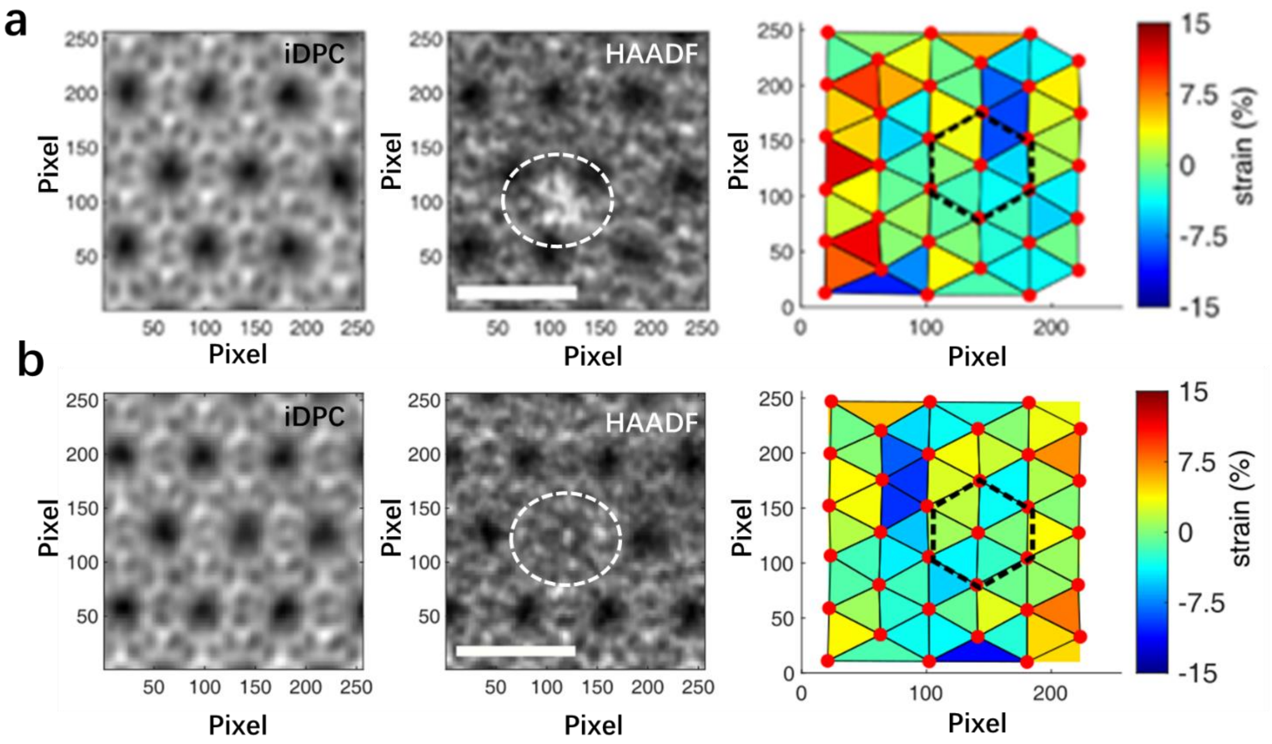

Supplementary Figure 24. Influence of the location of Ir clusters on the local strain. The iDPC and HAADF images are presented to show the size and location of the Ir clusters. The pixel size in this figure is 0.0174 nm and the scale bar is 2 nm.

As shown in the two sets of images, Ir clusters with similar sizes (~0.7 nm, corresponding to ~13 Ir atoms) give different local strain values because of their different location in the MWW zeolite structure. The Ir cluster shown in (a) is located at the 10MR window, showing an off-set corrected local absolute strain of 1.9% while the Ir cluster located at the 12MR supercage gives an off-set corrected local absolute strain of 0.2%.

The observed site-dependent local strain is consistent with the theoretical calculation results, which indicate a larger structural distortion and charge transfer is observed when Ir_{13} cluster is located at the 10MR window than that in the 12MR supercage.

Supplementary Note 8

The errors of the strain analysis with the practical metal-zeolite materials can be caused by multiple causes. Firstly, the strain analysis relies on the two-dimensional projection images of three-dimensional solid materials. In this sense, an Ir cluster observed in the HAADF image could be the overlapped image of several (for instance, two or three) Ir species at different depth. As a consequence, proximity between different Ir species can give rise to distortion values which surpass the range calculated for isolated ones. Likewise, the values determined in areas populated by Ir single atoms suggest a final charge state for these species which corresponds to interactions with the zeolite framework stronger than that determined for the vacuum-type DFT models.

Furthermore, as mentioned in the main text, the defective sites in the zeolite structure (for instance, the silanol groups), variation in the thickness of the MWW zeolite and the orientation of the zeolite will also influence the final iDPC image recorded. Though the extraction of the reference points in the experimental iDPC image proceeds automatically by algorithm, some error could be introduced due to the quality of the experimental iDPC image. In other words, the precision of our analysis methodology can be further improved by the application of more powerful detectors in the electron microscope.

Apart from the intrinsic errors of the method, additional factors could be at the roots of the observed deviations. Thus, the DFT analysis considered only cases of perfectly isolated Ir species. However, in the experimental images, concurrency of different species at nearby locations (either in the projection plane or along the projection direction) can take place, which could induce stronger structural distortions. In fact, the HAADF images of the two cases lying upscale (offset-corrected absolute strain values equal to 2.8 and 3.2) suggest the presence of more than one Ir atom in close vicinity.

References for supplementary information

1 Sang, X., Oni, A. A. & LeBeau, J. M. Atom column indexing: atomic resolution image analysis through a matrix representation. *Microsc Microanal* **20**, 1764-1771 (2014).

2 Kinyanjui, M. K. *et al.* Effects of electron beam generated lattice defects on the periodic lattice distortion structure in 1T−TaS2 and 1T−TaSe2 thin layers. *Physical Review B* **99** (2019).

3 Ning, S. *et al.* Scanning distortion correction in STEM images. *Ultramicroscopy* **184**, 274-283 (2018).

4 Lazic, I., Bosch, E. G. T. & Lazar, S. Phase contrast STEM for thin samples: Integrated differential phase contrast. *Ultramicroscopy* **160**, 265-280 (2016).

5 Mayoral, A., Anderson, P. A. & Diaz, I. Zeolites are no longer a challenge: atomic resolution data by aberration-corrected STEM. *Micron* **68**, 146-151 (2015).

6 Peters, J. J. *et al.* Artefacts in geometric phase analysis of compound materials. *Ultramicroscopy* **157**, 91-97 (2015).

7 Nord, M., Vullum, P. E., MacLaren, I., Tybell, T. & Holmestad, R. Atomap: a new software tool for the automated analysis of atomic resolution images using two-dimensional Gaussian fitting. *Adv Struct Chem Imaging* **3**, 9 (2017).

8 Zuo, J.-M. *et al.* Lattice and strain analysis of atomic resolution Z-contrast images based on template matching. *Ultramicroscopy* **136**, 50-60 (2014).

9 Yücelen, E., Lazić, I. & Bosch, E. G. T. Phase contrast scanning transmission electron microscopy imaging of light and heavy atoms at the limit of contrast and resolution. *Scientific Reports* **8** (2018).

10 Liu, L., Lopez-Haro, M., Calvino, J. J. & Corma, A. Tutorial: structural characterization of isolated metal atoms and subnanometric metal clusters in zeolites. *Nat Protoc* **16**, 1871-1906 (2021).

11 Carlsson, A., Alexandrou, I., Yücelen, E., Bosch, E. G. T. & Lazić, I. Low Dose Imaging Using Simultaneous iDPC- and ADF-STEM for Beam Sensitive Crystalline Structures. *Microscopy and Microanalysis* **24**, 122-123 (2018).

12 Liu, L. *et al.* Regioselective Generation of Single-Site Iridium Atoms and Their Evolution into Stabilized Subnanometric Iridium Clusters in MWW Zeolite. *Angew. Chem. Int. Ed.* **59**, 15695-15702 (2020).
